# Humanized biparatopic nanobody-based CAR-T cells overcome antigen-heterogeneity in multiple myeloma

**DOI:** 10.1186/s12967-026-08533-z

**Published:** 2026-06-29

**Authors:** Jincai Zhou, Kai Wu, Bixia Lei, Xinran Luo, Feifei Shi, Yuting Zhang, Xuefeng Kong, Huajing Wang, Joy Zhou, Hao Guo, Xiaowen He

**Affiliations:** OriCell Therapeutics Co. Ltd., Shanghai, 201203 China

**Keywords:** BCMA, CAR-T, Biparatopic, Multiple myeloma, Soluble BCMA, Antigen heterogeneity

## Abstract

**Background:**

Chimeric antigen receptor (CAR) T-cell therapy targeting B-cell maturation antigen (BCMA) shows activity in multiple myeloma (MM), yet relapse remains common owing to heterogeneous BCMA expression and soluble BCMA (sBCMA). Improving BCMA-directed CAR-T therapy requires persistence, enhanced antitumor activity, tolerance to low antigen density and resistance to sBCMA-mediated inhibition.

**Methods:**

We identified anti-BCMA VHHs from a humanized phage display library, constructed monospecific and biparatopic CARs, and evaluated their function and optimal designs in preclinical models, including patient-derived MM cells and xenografts.

**Results:**

Biparatopic Nab5822 CAR-T cells exhibited superior cytotoxicity and cytokine secretion (IL-2, IFN-γ, TNF-α) against antigen-heterogeneous MM cells in vitro, maintained activity under a supraphysiological sBCMA challenge at 120 ng/mL, and retained function under repeated exposure to clinically relevant sBCMA levels. Under repeated antigen challenge, Nab5822 preserved lysis, reduced exhaustion with an increased stem-cell memory T-cell compartment, and achieved durable tumor control in xenograft models without detectable toxicity. Mechanistic analyses indicated that the VHHs engage non-overlapping BCMA epitopes and promote improved immunological synapse organization together with coordinated proximal signaling, features that are not fully explained by equilibrium affinity alone and may contribute to sustained long-term T-cell function.

**Conclusions:**

Our findings support biparatopic CAR design as a strategy to tolerate low antigen density and resist sBCMA-mediated inhibition, providing a rationale for clinical evaluation of next-generation CAR T-cell therapies.

**Supplementary information:**

The online version contains supplementary material available at https://doi.org/10.1186/s12967-026-08533-z.

## Introduction

Despite therapeutic advances over the past two decades, multiple myeloma (MM) remains largely incurable [1]. Consequently, developing therapeutic approaches that can induce durable tumor regression and improve disease outcomes is urgently needed, particularly in high-risk relapsed and refractory multiple myeloma (RRMM) [2]. Chimeric antigen receptor (CAR) T-cell therapy has transformed the management of hematologic malignancies and substantially improved response and survival in RRMM [3, 4]. CAR-T cells are antigen-directed therapies that recognize tumor-associated antigens (TAAs) independently of MHC class I (MHC-I) and typically comprise an extracellular antigen-recognition domain, a hinge and transmembrane region, an intracellular activation domain and a co-stimulatory motif [5]. As of June 2025, seven CAR-T therapies had been approved by the US Food and Drug Administration (FDA) for specific forms of leukemia, lymphoma, and myeloma [6]. Despite these successes, several obstacles limit durable remissions [7, 8].

B-cell maturation antigen (BCMA) is an attractive target for CAR-T therapy due to its restricted expression on normal plasma cells and prevalent expression on MM cells [9]. BCMA is a 184-amino acid (aa) transmembrane protein composed of a 54-aa extracellular domain, a 23-aa transmembrane segment and a 107-aa intracellular tail [10]. Cleavage by the multisubunit γ-secretase complex at Trp57 releases sBCMA, reducing cell-surface antigen density and impairing CAR-T cell functionality [11]. Clinical studies indicate that CAR-T efficacy is closely associated with antigen density, with a minimum threshold necessary to sustain T-cell activity, particularly in patients receiving CD20-, CD22-, or B7-H3-directed CAR-T therapies [12–14]. Concomitantly, accumulation of sBCMA in patient plasma/BM can act as a decoy for BCMA-binding agents and limit therapeutic efficacy [15]. Clinical evidence indicates that sBCMA can function as an antigen sink, reducing CAR T-cell target recognition and associating with clinical outcomes in anti-BCMA CAR T-cell therapy [15, 16].

In pivotal trials, idecabtagene vicleucel (ide-cel, KarMMa) achieved an overall response rate (ORR) of 73% with a median progression-free survival (mPFS) of 8.8 months, whereas ciltacabtagene autoleucel (cilta-cel, CARTITUDE-1) reached an ORR of 97.9% and an mPFS of 34.9 months [17, 18]. Nevertheless, 1-year progression rates of 60% for ide-cel and 33% for cilta-cel indicate that relapse remains a challenge for all approved anti-BCMA CAR-T products [17]. Heterogeneous or reduced BCMA expression and interference by sBCMA are recognized mechanisms of relapse that can impair CAR-T recognition and function [19]. These observations motivate strategies to enhance CAR-T sensitivity to low antigen density and to mitigate sBCMA-mediated inhibition.

To address these challenges, various strategies have been explored to optimize BCMA-specific CAR-T cells. These include incorporation of additional chimeric co-stimulatory receptors (CCRs) [20], tandem CAR designs targeting both CD38 and BCMA or GPRC5D and BCMA [21, 22], HLA-independent T cell (HIT) receptors [23], combination therapies with immune checkpoint inhibitors (ICIs) or γ-secretase inhibitors (GSIs) to prevent sBCMA shedding [24, 25], reinforcement with endogenous cytokines [26], and structural optimization of hinge, transmembrane, and co-stimulatory domains to enhance CAR-T functionality [27].

Here, we screened a humanized VHH phage-display library and prioritized candidate VHHs on the basis of affinity and T-cell functional screening, selecting two lead VHHs for CAR development. To develop a functional layout and optimize CAR-T structures for MM treatment, we generated monospecific and bispecific BCMA CARs and evaluated their performance in vitro and in murine xenograft models. The biparatopic Nab5822 CAR-T cells demonstrated superior cytotoxicity and cytokine secretion against antigen-heterogeneous MM cells, even in the presence of high sBCMA concentrations. Nab5822 CAR-T cells maintained stem-cell-memory (T_SCM_) phenotypes, displayed reduced exhaustion marker expression and showed low antigen-independent (tonic) signalling ex vivo. In xenograft models with low antigen density, biparatopic Nab5822 CAR-T cells conferred durable tumor control and prolonged survival without overt toxicity. Mechanistic analyses mapped two non-overlapping BCMA epitopes recognized by the VHHs and showed that biparatopic engagement improves immunological synapse organization and coordinates proximal CD3ζ/STAT5 signaling, thereby potentially contributing to the enhanced activity of Nab5822. Collectively, these data provide a rationale for biparatopic CAR design to overcome antigen-heterogeneity and sBCMA-mediated inhibition, supporting the development of next-generation CAR-T therapies for MM.

## Results

### Identification of VHHs with high affinity and specificity for human BCMA

**Fig. 1:**
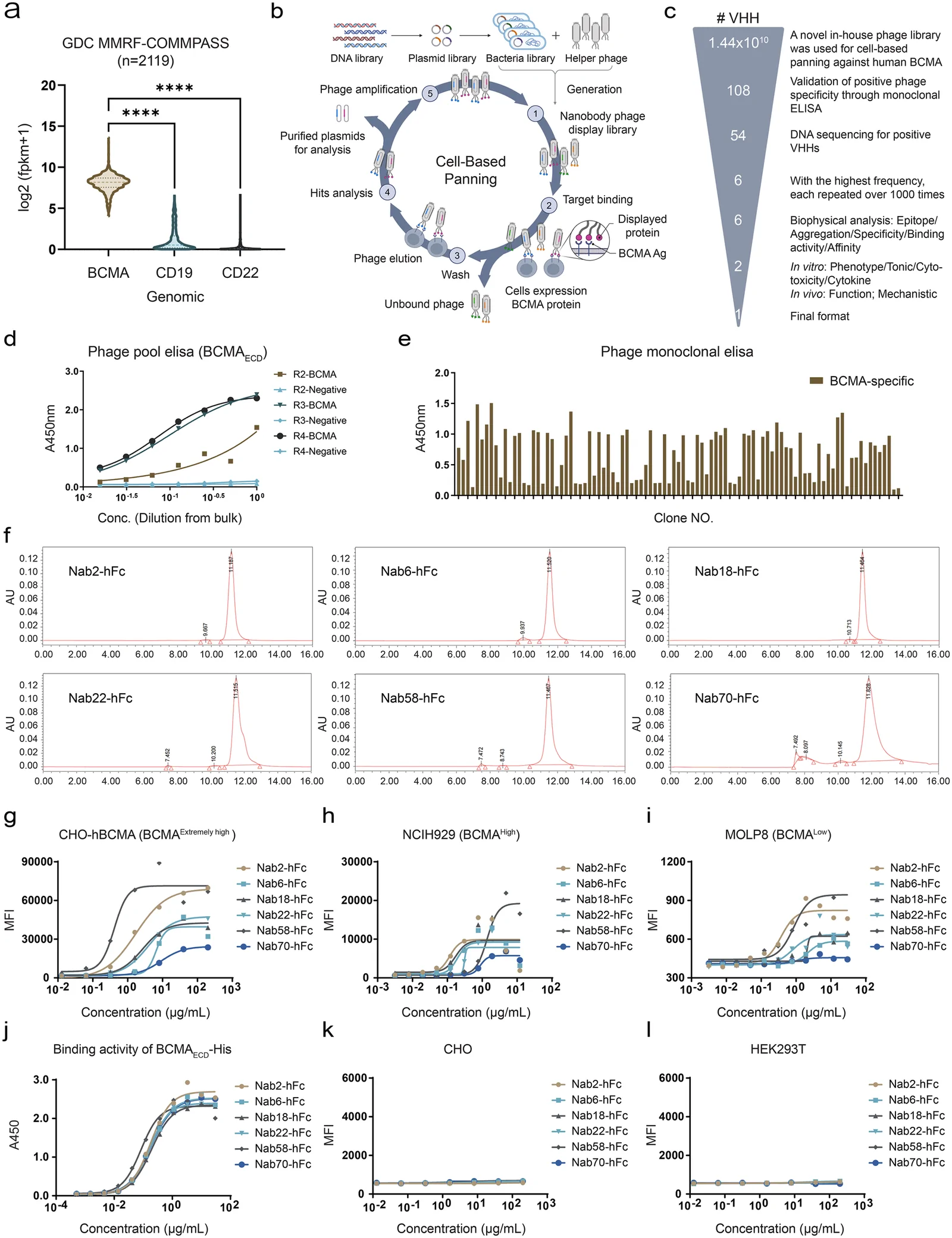
Discovery of high affinity and specific VHHs against human BCMA. (**a**) A total of 2119 samples from MM patients were immunostained with antibodies targeting BCMA, CD19, and CD22. Each data point on the graph corresponds to an individual patient’s sample, and the analysis was conducted using the UCSC Xena platform. (**b**) Phage-display library screening strategy. The process includes DNA library generation, biopanning, and identification of specific binders through iterative selection and analysis. (**c**) A diagram of the panning procedure, six candidate VHHs were selected for further study. (**d**) Polyclonal analysis of dilutions of the phage pool from each round of biopanning by ELISA. (**e**) Monoclonal analysis of rescued phage from randomly selected clones from rounds 3 and 4 of biopanning by ELISA. (**f**) The purity of six candidate VHHs was evaluated using HPLC-SEC. (**g-i**) Flow cytometry analysis of the binding activity of six candidate VHHs to target cells with heterogeneous BCMA expression. (**j**) ELISA analysis of the binding activity of six candidate VHHs to BCMA_ECD_-His antigen. (**k-l**) Flow cytometry analysis of the binding activity of six candidate VHHs to BCMA-negative cell lines CHO and HEK293T. Data were acquired using a BD Biosciences FACS flow cytometer and analyzed with FlowJo software

BCMA contains an extracellular domain of 54-aa stabilized by disulfide bonds, providing structural basis for its role in multiple myeloma pathogenesis (Supplementary Fig. 1a-b). Genomic analyses of MM patient samples demonstrate that BCMA expression significantly exceeds levels of CD19 and CD22, establishing BCMA as a superior therapeutic target for this disease (Fig. 1a). To identify VHHs with high affinity and specificity for human BCMA, we engineered CHO-hBCMA and HEK293T-hBCMA cell lines stably over-expressing human BCMA via lentiviral transduction of the parental cell lines. Using these cell lines, we screened VHHs from an in-house phage display-based synthetic nanobody library (Fig. 1b–c). Supernatants from biopanning phage pools showed a progressive increase in binding to BCMA_ECD_, CHO-hBCMA, and HEK293T-hBCMA from rounds 2 to 4, as confirmed by polyclonal phage ELISA (Fig. 1d and Supplementary Fig. 1c-d). Potential phage monoclonal candidates were screened for high binding activity to BCMA using ELISA (Fig. 1e). Through high-throughput monoclonal sequencing, we identified the top 6 distinct clones with the highest frequency, exceeding 1,000 repetitions. Accordingly, we constructed and purified the corresponding antibodies for further analysis. HPLC-SEC and SDS-PAGE confirmed their high purity, demonstrating their suitability for subsequent validation (Fig. 1f and Supplementary Fig. 1e-f).

To confirm the binding affinity and specificity of the identified VHHs, we performed flow cytometry, ELISA, and SPR using recombinant BCMA_ECD_-His protein. BCMA-positive cell lines, including CHO-hBCMA, NCIH929, and MOLP8, which display varying levels of BCMA expression, along with a panel of BCMA-negative cell lines from diverse tissue origins, were tested to ensure specificity. We found that six candidate VHHs exhibited high binding activity for BCMA-positive cell lines and BCMA_ECD_-His protein in a dose-dependent manner (Fig. 1g–j and Supplementary Fig. 1 g), with no detectable binding to BCMA-negative cell lines (Fig. 1k–l and Supplementary Fig. 2a-l). Based on their binding affinity and specificity for BCMA, six candidate VHHs were selected for CAR generation.

### Identification of VHHs for functional BCMA CAR-T cells

**Fig. 2:**
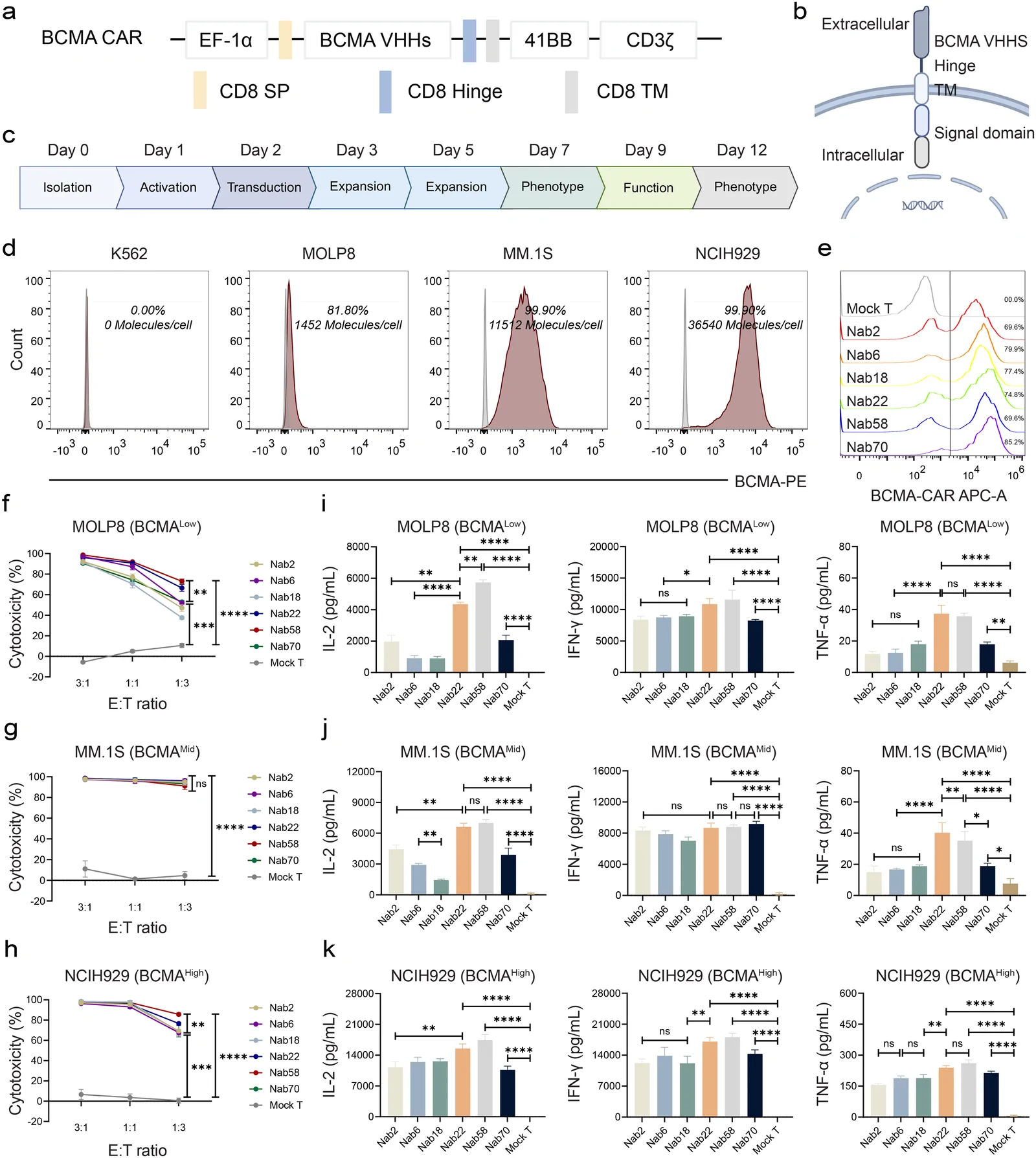
Functional screening of VHHs for BCMA CAR-T cells. (**a**) Schematic diagram of the recombinant lentiviral vectors encoding BCMA-targeted VHH CARs generated in this study. (**b**) Schematic diagram of the BCMA-CAR-T construct, comprising a BCMA-specific VHH, CD8α hinge and transmembrane domains, and a 4-1BB/CD3ζ co-stimulatory signaling module. (**c**) Workflow of the step-based CAR-T assay, illustrating the sequential steps for functional validation and activity assessment of CAR-T cells. (**d**) Quantification of heterogeneous BCMA expression on K562, MOLP8, MM.1S, and NCIH929 cells using BD Quantibrite beads. Molecule numbers per cell are 0, 1,452, 11,512, and 36,540, respectively. (**e**) Surface expression of VHH BCMA CARs on T cells 72 hours after lentiviral transduction, measured by flow cytometry. (**f-h**) Cytotoxicity of six VHH CAR-T cells and mock T cells against MOLP8 (BCMA^Low^), MM.1S (BCMA^Mid^), and NCIH929 (BCMA^High^) target cells at E:T ratios of 3:1, 1:1, and 1:3. (**i-k**) Cytokine production was measured by ELISA for IL-2, IFN-γ, and TNF-α in supernatants collected after co-culture at an E:T ratio of 1:1. Data are presented as mean ± SD. Mock T cells served as negative controls. Statistical significance was determined by one-way ANOVA, followed by Dunn’s multiple comparisons test; ns, not significant; *p < 0.05, **p < 0.01, ***p < 0.001, ****p < 0.0001

To identify VHHs suitable for functional CAR-T cells, we individually engineered CAR constructs incorporating the six candidate VHHs specific for human BCMA for initial evaluation. All CARs were generated with VHHs in-frame, incorporating the hinge and transmembrane domains of CD8α, along with the cytoplasmic domains of 4-1BB and CD3ζ (Fig. 2a–b). The stepwise workflow for functional validation of CAR-T cells is shown (Fig. 2c). Antigen escape or downregulation is a well-established mechanism of resistance to immunotherapies [28]. To evaluate CAR-T activity across varying BCMA expression levels, we selected multiple MM cell lines exhibiting heterogeneous BCMA expression, with K562 cells serving as a negative control (Fig. 2d). All VHH-based CAR-T cells proliferated upon stimulation with anti-CD3/CD28, with Nab58, Nab22, and Nab70 CAR-T cells showing similarly high expansion capacity and cell viability (Supplementary Fig. 3a-b). On day 9, in the absence of antigen stimulation, T cell volumes in Nab2, Nab6, and Nab18 were slightly higher than those in Nab22, Nab58, and Nab70, consistent with increased antigen-independent baseline activation that is commonly associated with tonic signaling and may delay the return to a resting state (Supplementary Fig. 3c). Seventy-two hours post-transduction, flow cytometry showed that VHH-based CAR-T cells exhibited similar transduction efficiencies, ranging from 69.6% to 85.2% (Fig. 2e). Additionally, flow cytometry revealed that VHH-based CAR-T cells exhibited CD4/CD8 ratios comparable to those of mock T cells (Supplementary Fig. 3d-e). Following a 24-hour co-culture, all VHH-based CAR-T cells efficiently lysed MM cell lines with heterogeneous BCMA expression across multiple E:T ratios. Nab22 and Nab58 CAR-T cells showed the highest activity at an E:T ratio as low as 1:3 in MOLP8 and NCH929 cells (Fig. 2f–h). Comparable results were observed in co-cultures containing 120 ng/mL sBCMA from the same donor (Supplementary Fig. 4a-c), and consistent findings were obtained with CAR-T cells derived from different donors (Supplementary Fig. 4d-f). Furthermore, compared with mock T cells, all VHH-based CAR-T cells secreted the proinflammatory cytokines IFN-γ, TNF-α, and IL-2 in response to MM cell lines exhibiting heterogeneous BCMA expression at a 1:1 E:T ratio, with Nab22 and Nab58 CAR-T cells producing higher cytokine levels than the others (Fig. 2i–k and Supplementary Fig. 4 g-l). Together, these data demonstrate that Nab22 and Nab58 CAR-T cells exhibit enhanced cytotoxicity against BCMA-positive cells, accompanied by increased secretion of cytotoxic factors.

### Sustained function and memory-like phenotypes of VHH-based BCMA CAR-T cells

**Fig. 3:**
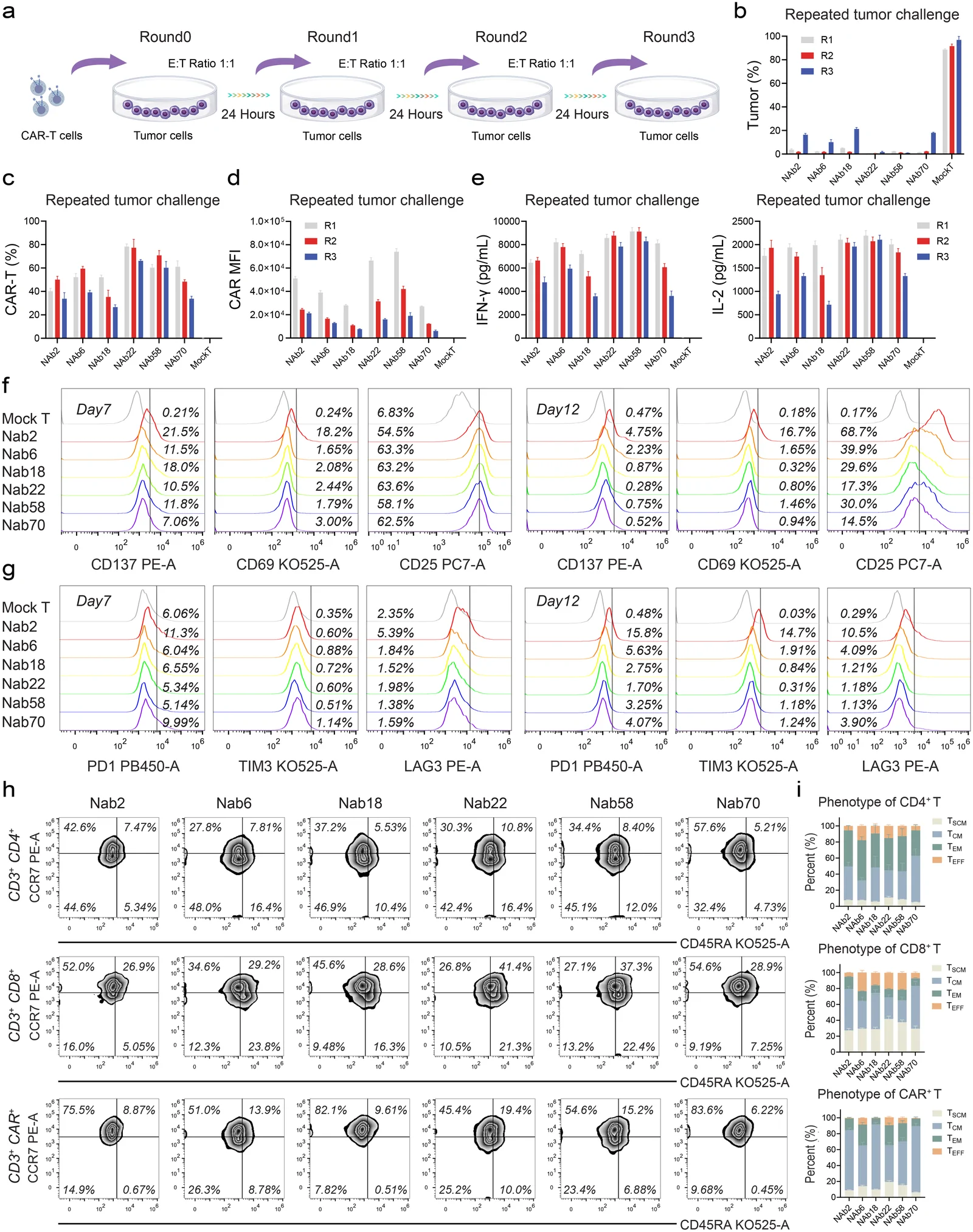
Characterization of VHH-based BCMA CAR-T cells. (**a**) Schema of the multi-round repeated antigen stimulation experiment. Tumor cells were seeded in 12-well plates, and CAR-T cells were added at an E:T ratio of 1:1. Every 24 hours, cells were collected, washed with PBS, resuspended in fresh X-VIVO medium, and counted using AO/PI reagent. Cells were then analyzed by flow cytometry, and culture supernatants were collected for cytokine measurement by ELISA. (**b-d**) Six VHH CAR-T and mock T cells were separately co-cultured with tumor cells over successive rounds. Residual tumor cells (**b**), CAR-T cell numbers (**c**), and CAR MFI (**d**) were quantified after each round. (**e**) Concentrations of IFN-γ and IL-2 in supernatants after co-culture with tumor cells were measured by ELISA. (**f**) Flow cytometry analysis and quantitative summary of activation markers CD137 (4-1BB), CD25, and CD69 on CAR-T cells on Day 7 and Day 12 of culture. (**g**) Flow cytometry analysis and quantitative summary of exhaustion markers PD-1, TIM-3, and LAG-3 on CAR-T cells on Day 7 and Day 12 of culture. (**h**) Representative dot plots show the phenotype of CD4^+^, CD8^+^, and CAR^+^ T cells. Cells were stained with CCR7 and CD45RA antibodies and assigned to subsets as stem cell memory (T_SCM_, CCR7^+^/CD45RA^+^), central memory (T_CM_, CCR7^+^/CD45RA^-^), effector memory (T_EM_, CCR7^-^/CD45RA^-^), and effector (T_EFF_, CCR7^-^/CD45RA^+^) cells. Gating boundaries were defined using CCR7-FMO and CD45RA-FMO controls. (**i**) Quantification of T cell phenotypes. Frequencies of stem cell memory, central memory, effector memory, and effector subsets were determined for CD4^+^, CD8^+^, and CAR^+^ T cells based on the representative flow cytometry plots in (**h**)

In addition to cytotoxicity and cytokine assays, residual tumor burden, BCMA CAR surface expression, MFI, and cytokine secretion were monitored during repeated antigen stimulation. In the repeated antigen stimulation assay, all VHH-based CAR-T cells exhibited robust cytotoxicity against MM.1S cells, with Nab22 and Nab58 CAR-T cells showing higher killing potential (Fig. 3a–b). Nab22 and Nab58 CAR-T cells also displayed higher frequencies of BCMA CAR-T cells and sustained relatively high CAR MFI following chronic antigen stimulation (Fig. 3c–d). Additionally, ELISA results demonstrated that co-culture with MM.1S cells induced IL-2 and IFN-γ production in all VHH-based CAR-T cells compared with mock T cells. Notably, Nab22 and Nab58 CAR-T cells secreted higher levels of IFN-γ and IL-2 than the other four CAR-T groups upon MM.1S stimulation (Fig. 3e). Similar trends were observed using MOLP8 cells as targets (data not shown).

Moreover, we profiled CD3^+^, CD4^+^, CD8^+^, and CAR^+^ T-cell subsets throughout the culture period to characterize their in vitro phenotypic evolution. The expression of activation markers CD137, CD25, and CD69 was marginally lower in Nab22, Nab58, and Nab70 CAR-T cells than in Nab2, Nab6, and Nab18 cells, with a comparatively larger difference observed for the late activation marker CD25 on day 12, particularly relative to Nab2 and Nab6 (Fig. 3f). Excessive expression of activation markers during the late stages of CAR-T culture can induce apoptosis and compromise long-term persistence [29].

Quantitative analysis of exhaustion markers PD1, TIM3, and LAG3 highlighted modest but discernible differences among CAR-T groups and between Day 7 and Day 12 (Fig. 3g). In particular, Nab18, Nab22, and Nab58 CAR-T cells showed relatively lower exhaustion-marker expression, especially on Day 12, which may be consistent with their superior antitumor activity observed in other assays (Fig. 2f–k and Supplementary Fig. 4a-l). Furthermore, Nab22 and Nab58 CAR-T cells exhibited an increased proportion of stem cell memory T cells (T_SCM_, CCR7^+^/CD45RA^+^) across CD4^+^, CD8^+^, and BCMA CAR^+^ populations compared with other CAR-T cells (Fig. 3h–i), suggesting enhanced expansion, differentiation, and self-renewal potential upon MM cell stimulation [30, 31]. This memory-like phenotype likely contributes to the sustained cytotoxicity observed during repeated antigen exposure, consistent with the killing observed under serial stimulation (Fig. 3b–e). Collectively, these in vitro results support the prioritization of Nab22 and Nab58 for further BCMA-specific CAR-T optimization and their progression into preclinical and clinical studies.

### Biparatopic Nab5822 CAR-T cells demonstrate superior functionality in vitro

**Fig. 4:**
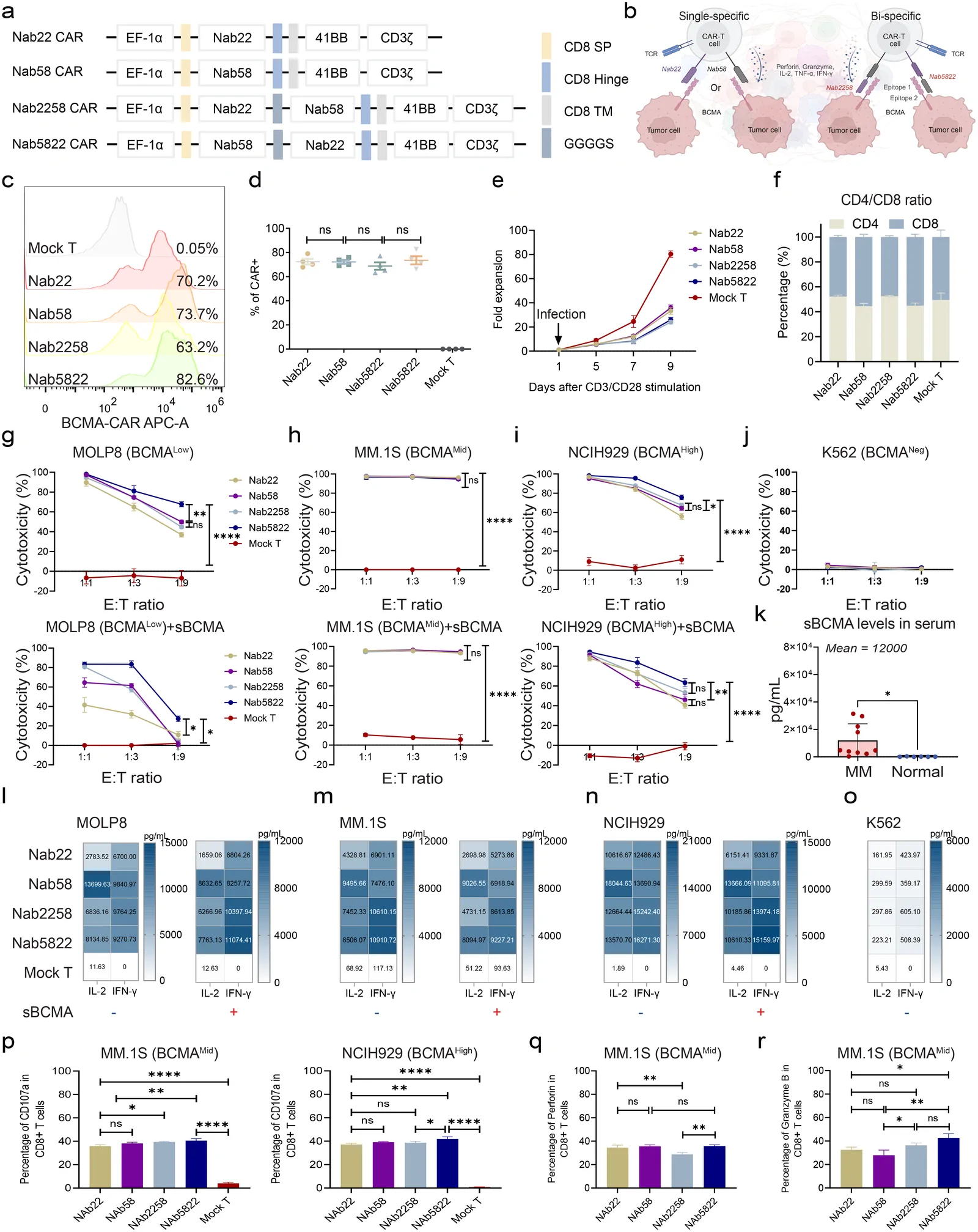
Enhanced in vitro activity of biparatopic Nab5822 CAR-T cells. (**a**) Schematic of recombinant lentiviral vectors encoding monospecific BCMA CAR, bispecific Nab2258 CAR, and Nab5822 CAR. (**b**) A schematic illustrating the potential mechanisms of action of monospecific BCMA CAR, bispecific Nab2258 CAR, and Nab5822 CAR-T cells against tumor cells. (**c-d**) Surface BCMA CAR expression on T cells 72 hours after lentiviral transduction was measured by flow cytometry, with representative plots shown in (**c**) and quantification across four donors in (**d**). (**e**) Total expansion Fold of CAR-T cells cultured in vitro with anti-CD3/CD28 stimulation at the indicated time points. (**f**) Percentages of CD4 and CD8 subtypes in CAR-T and mock T cells. (**g-j**) Cytotoxicity of CAR-T and mock T cells against MOLP8 (BCMA^Low^) (**g**), MM.1S (BCMA^Mid^) (**h**), NCIH929 (BCMA^High^) (**i**), and K562 (BCMA^Neg^) (**j**) target cells at E:T ratios of 1:1, 1:3, and 1:9, shown in top panels without sBCMA and bottom panels with sBCMA. Data are presented as mean ± SD of triplicate wells. (**k**) Serum soluble BCMA levels in 10 MM patients were measured by ELISA, with a mean concentration of 12,000 pg/mL. (**l-o**) Representative heat maps illustrating cytokine production of CAR-T and mock T cells against MOLP8 (BCMA^Low^) (**l**), MM.1S (BCMA^Mid^) (**m**), NCIH929 (BCMA^High^) (**n**), and K562 (BCMA^Neg^) (**o**) target cells at E:T ratios of 1:1, measured by CBA. Quantitative data are shown for the indicated cytokines in the absence or presence of sBCMA. (**p**) Degranulation of CD8^+^ T cells was assessed by surface CD107a expression using flow cytometry after 4 h of co-culture at an E:T ratio of 1:1. (**q-r**) Intracellular expression of perforin (**q**) and granzyme B (**r**) In CD8^+^ T cells was assessed by flow cytometry after 4 h of co-culture at an E:T ratio of 1:1. Statistical significance was determined by one-way ANOVA, followed by Dunn’s multiple comparisons test, or by an unpaired t-test; ns, not significant; *p < 0.05, **p < 0.01, ***p < 0.001, ****p < 0.0001

Using Nab22 and Nab58, we generated bispecific CAR-T constructs incorporating two anti-BCMA VHHs into 4-1BB based formats for subsequent in vitro and in vivo evaluation (Fig. 4a–b). In our previous work, we generated and tested homo-bivalent VHH tandems; however, despite the expected increase in apparent binding strength, these constructs did not yield the anticipated functional enhancement. Based on these observations, we therefore did not include homo-bivalent tandem controls in the present study; instead, we focused on tandems built from non-identical VHHs to evaluate the impact of epitope diversity. All CAR-T cells exhibited comparable BCMA-CAR transduction efficiencies and stable expression in T cells from four independent donors (Fig. 4c–d). Although all CAR-T cells showed lower fold expansion than mock T cells under anti-CD3/CD28 stimulation, all CAR-T-cell products remained expandable in vitro (Fig. 4e). Nab58 and Nab5822 CAR-T cells showed a trend toward a higher CD8^+^ T-cell proportion compared with Nab22 and Nab2258 (Fig. 4f). Although a higher CD8+ T-cell proportion has been associated with enhanced antitumor activity in some settings [32], given the known influence of donor starting material and culture conditions on CD4/CD8 composition, we cannot attribute this variation solely to the CAR construct.

We next evaluated the cytotoxicity of different CAR-T cells against a panel of MM cell lines, including MOLP, MM.1S, and NCIH929, at E:T ratios of 1:1, 1:3, and 1:9, with the primary aim of comparing the relative performance of each CAR design within the same target-cell context. At both E:T ratios of 1:3 and 1:9, Nab5822 CAR-T cells efficiently lysed MM cells with heterogeneous BCMA expression, even in the presence of 120 ng/mL sBCMA, an intentionally supraphysiologic challenge condition corresponding to approximately 10-fold the mean serum concentration measured in our patient cohort (Fig. 4g–i and k), while sparing BCMA-negative K562 cells (Fig. 4j). Similar results were observed with CAR-T cells derived from an additional patient with R/R MM who had received at least three prior lines of therapy (Supplementary Fig. 5a-b). These findings indicate that Nab5822 CAR-T cells exhibit potent antitumor activity across MM cell lines with heterogeneous BCMA expression. Notably, the apparently higher susceptibility of MM.1S cells relative to NCIH929 cells, despite their lower BCMA expression, suggests that BCMA surface density alone does not fully determine target-cell killing and that cell-intrinsic differences in target-cell sensitivity may also contribute to the observed pattern. Concurrently, we assessed cytokine secretion by CAR-T cells after 24 h of co-culture with MM cell lines at a 1:1 E:T ratio. All CAR-T cells except Nab22 specifically secreted comparable levels of IL-2, TNF-α, and IFN-γ, whereas mock T cells produced minimal cytokines (Fig. 4l–o and Supplementary Fig. 5c). Importantly, high concentrations of sBCMA did not significantly impair cytokine production by Nab5822 CAR-T cells, indicating preserved functional activation despite potential antigen blockade (Fig. 4l–o and Supplementary Fig. 5d). Supplementary Fig. 5c-d shows additional cytokine measurements from the same donor and co-culture assay, obtained simultaneously to complement the main cytokine dataset. Additionally, Nab5822 CAR-T cells showed spontaneous IFN-γ secretion comparable to that of comparator CAR-T cells, indicating that they did not exhibit overt antigen-independent hyperactivation (Fig. 4o). Consistent with this observation, a Jurkat-NFAT-EGFP reporter assay showed that Nab5822 exhibited basal NFAT activation comparable to that of comparator CARs in the absence of target cells, while maintaining comparable inducible signaling upon target-cell engagement (Supplementary Fig. 6a-b).

Furthermore, we examined degranulation and cytotoxic potential by measuring CD107a, perforin, and granzyme B expression on CD8^+^ T cells. In MM.1S cells, Nab5822 CAR-T cells induced CD107a expression in ~40% of CD8^+^ T cells, which was significantly higher than that observed in Nab22 or mock T cells (*p* < 0.01), whereas Nab58 and Nab2258 CAR-T cells showed comparable levels (Fig. 4p and Supplementary Fig. 6c). In NCIH929 cells, Nab5822 CAR-T cells elicited the highest CD107a response (~42%, *p* < 0.0001 vs. mock T), which was significantly higher than that of Nab22 and Nab2258 CAR-T cells (Fig. 4p and Supplementary Fig. 6d). Consistently, significant differences in perforin (Fig. 4q) and granzyme B (Fig. 4r) expression were observed among CD8^+^ T cells in all CAR-T groups. Collectively, these results indicate that Nab5822 CAR-T cells exhibit superior cytotoxicity compared with monospecific CAR-T cells.

### Phenotypic characterization of Nab5822 CAR-T cells

To ensure clinical relevance, it is essential to evaluate CAR-T cell function in patient-derived T cells rather than relying exclusively on healthy donor T cells, which differ substantially in activation status, exhaustion profile, and metabolic fitness [33]. Nab5822 CAR-T cells maintained effective cytotoxic activity across diverse patient-derived T cells, despite impaired baseline conditions in patients who had undergone more than three prior lines of therapy at enrollment (Supplementary Fig. 7a-h). In addition, Nab5822 CAR-T cells showed low tonic signaling, as reflected by low spontaneous IFN-γ secretion in the absence of antigen stimulation, a feature associated with reduced exhaustion and enhanced persistence, indicating that functional Nab5822 CAR-T cells can be effectively generated from patient-derived PBMCs (Supplementary Fig. 7 h).

**Fig. 5:**
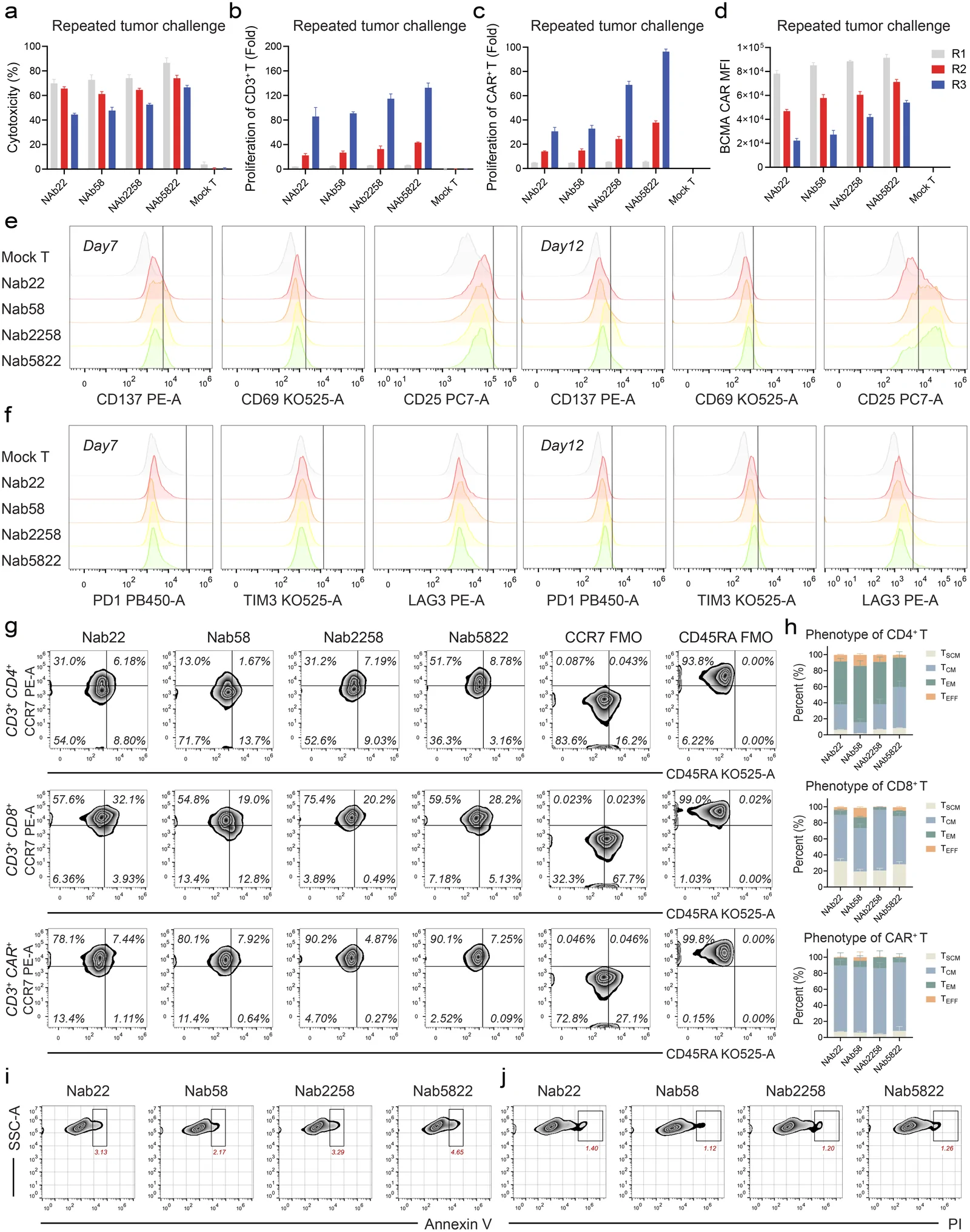
Persistence and proximal signaling of Nab5822 CAR-T cells. (**a-d**) CAR-T and mock T cells were separately co-cultured with tumor cells over successive rounds, with cytotoxicity (**a**), CD3^+^ T cell numbers (**b**), CAR-T cell numbers (**c**), and CAR MFI (**d**) quantified after each round. For (**b**) and (**c**), the Fold expansion was calculated as a cumulative proliferation index across successive rounds. (**e**) Flow cytometry analysis of activation markers CD137, CD25, and CD69 on CAR-T cells on Day 7 and Day 12 of culture. (**f**) Flow cytometry analysis of exhaustion markers PD-1, TIM-3, and LAG-3 on CAR-T cells on Day 7 and Day 12 of culture. (**g**) Representative dot plots show the phenotype of CD4^+^, CD8^+^, and CAR^+^ T cells. Cells were stained with CCR7 and CD45RA antibodies and assigned to subsets as stem cell memory (T_SCM_, CCR7^+^/CD45RA^+^), central memory (T_CM_, CCR7^+^/CD45RA^-^), effector memory (T_EM_, CCR7^-^/CD45RA^-^), and effector (T_EFF_, CCR7^-^/CD45RA^+^) cells. (**h**) Quantification of T cell phenotypes. Frequencies of stem cell memory, central memory, effector memory, and effector subsets were determined for CD4^+^, CD8^+^, and CAR^+^ T cells based on the representative flow cytometry plots in (**g**). (**i-j**) Representative flow cytometry plots showing CAR-T cell apoptosis, assessed by Annexin V (**i**) and propidium iodide (PI, **j**) staining. Statistical significance was determined by one-way ANOVA, followed by Dunn’s multiple comparisons test, or by an unpaired t-test; ns, not significant; *p < 0.05, **p < 0.01, ***p < 0.001, ****p < 0.0001

Long-term persistence is critical for sustained CAR-T-cell efficacy [34, 35]. Using a serial co-culture assay in which effector cells encountered fresh tumor cells every 24 hours, thereby mimicking dynamic in vivo killing, Nab5822 CAR-T cells sustained ≥ 90% specific lysis and preserved proliferative capacity across three consecutive cycles. Notably, in the presence of 12 ng/mL sBCMA, corresponding to the mean serum concentration measured in our patient cohort, Nab5822 CAR-T cells retained superior killing activity and proliferative capacity (Supplementary Fig. 8a-c). These findings indicate that Nab5822 remains functionally active under repeated exposure to clinically relevant sBCMA levels (Fig. 5a–d and Supplementary Fig. 8a-c). Activation profiling of CD137, CD25, and CD69 revealed that Nab5822 CAR-T cells did not induce a hyperactivated state during culture and remained stable or decreased between day 7 and day 12 (Fig. 5e). Consistently, exhaustion profiling of PD1, TIM3, and LAG3, key mediators of T-cell dysfunction upon antigen stimulation, revealed that Nab5822 CAR-T cells maintained low exhaustion levels during culture, with LAG3 expression remaining notably low (Fig. 5f). Moreover, Nab5822 CAR-T cells exhibited a higher fraction of stem cell memory T cells (T_SCM_, CCR7^+^/CD45RA^+^) across CD4^+^, CD8^+^, and BCMA CAR^+^ subsets compared with Nab2258 CAR-T cells on day 7 (Fig. 5g–h), implying greater expansion, differentiation, and self-renewal potential [30, 31]. Similar trends were observed on day 12 (Supplementary Fig. 8d-e). Apoptosis analysis further confirmed that Nab5822 CAR-T cells did not exhibit elevated apoptosis relative to other CAR-T cells, as evidenced by comparable percentages of Annexin V- and PI-positive cells (*p* > 0.05, Fig. 5i–j and Supplementary Fig. 8f-g).

Consistent with established proximal CAR and TCR signaling kinetics [36, 37], Nab5822 CAR-T cells exhibited rapid CD3ζ phosphorylation within 15 min of tumor engagement that remained detectable at 30 min. STAT5 phosphorylation increased between 15 and 30 min, suggesting coordinated proximal and downstream signaling following antigen recognition [38] (Supplementary Fig. 8 h-k). Taken together, these results demonstrate that bispecific Nab5822 CAR-T cells maintain robust cytotoxicity, low exhaustion, preserved memory phenotypes, minimal apoptosis, and a coordinated proximal signaling cascade, supporting their enhanced persistence and antitumor potential.

### Biparatopic Nab5822 CAR-T cells demonstrate superior antitumor activity and safety in vivo

**Fig. 6:**
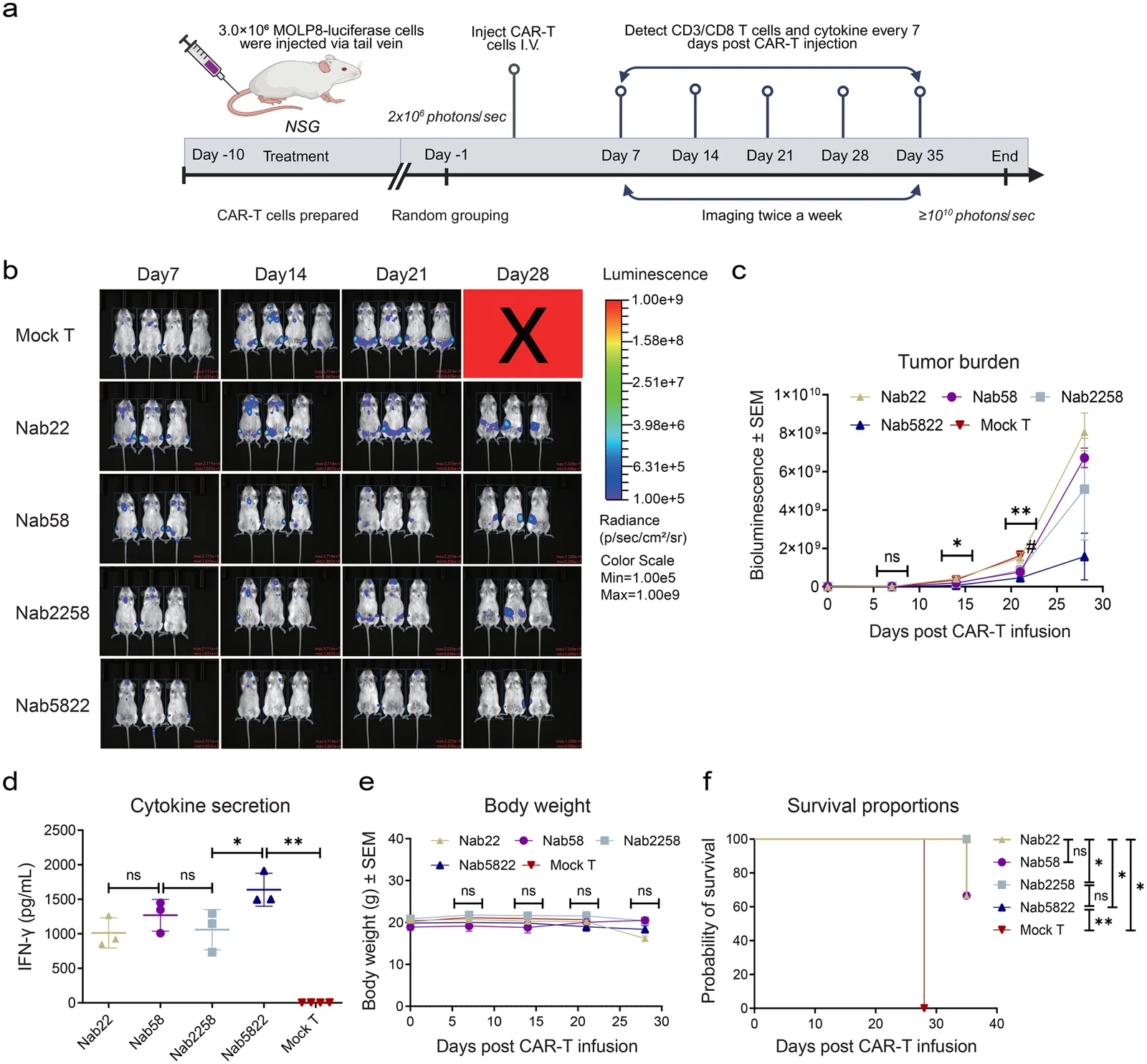
Enhanced in vivo antitumor activity of Nab5822 CAR-T cells. (**a**) Schematic of the xenograft model used to assess CAR-T cell activity in vivo. NSG mice received 3.0 × 10^6 MOLP8-luc cells on day −10 and 2.0 × 10^6 cryopreserved CAR-T or mock T cells on day 0. (**b**) Total flux represents the tumor burden in MOLP8-luc bearing mice at the indicated time points (*n* = 3–4 biological replicates). (**c**) Bioluminescence was monitored during treatment (*n* = 3–4 biological replicates). Mice marked with # were euthanized upon reaching the humane endpoint because tumor burden exceeded 1.0 × 10^10 photons/s. Data are presented as mean ± SEM. (**d**) IFN-γ secretion levels in each group were measured by ELISA on day 7 after infusion of CAR-T or mock T cells. Data are presented as mean ± SD. (**e**) in vivo toxicity was assessed by monitoring mouse body weight during treatment. Data are presented as mean ± SEM. (**f**) Kaplan-Meier survival curves of MOLP8-Luc bearing mice treated as described in (**b, c**). Statistical significance was determined by one-way ANOVA followed by Dunn’s multiple comparisons test, two-way ANOVA followed by Tukey’s multiple comparisons test, or log-rank test

To evaluate the antitumor activity of Nab5822 CAR-T cells in vivo, we employed MM xenograft models using nonobese diabetic scid gamma (NSG) mice. A total of 3.0 × 10^6 MOLP8-Luciferase cells were injected via the tail vein. Tumor engraftment was confirmed by baseline bioluminescence imaging (BLI) on day 10 post-injection. Mice were randomly assigned to control or treatment groups and received a single intravenous injection of CAR-T or mock T cells (2.0 × 10^6 cells). Tumor burden was monitored twice weekly by BLI, and mice were observed daily for signs of toxicity, including weight loss, lethargy, or abnormal behavior (Fig. 6a).

As expected, Nab5822 CAR-T cells demonstrated superior antitumor efficacy compared with all other groups (Fig. 6b–c). IFN-γ secretion by Nab5822 cells at day 7 post-injection was significantly higher (1496–1913 pg/mL) than that observed in the Nab22 (851–1263 pg/mL), Nab58 (1010–1450 pg/mL), and Nab2258 (735–1297 pg/mL) groups (Fig. 6d), consistent with in vitro data (Fig. 4l–o). No significant body weight loss was observed in any treatment group, indicating a favorable safety profile (Fig. 6e). Additionally, kaplan-Meier survival analysis revealed significantly prolonged survival in mice treated with Nab5822 CAR-T cells, with overall survival exceeding 35 days compared with 28 days in the mock T cell group (Fig. 6f). Collectively, these in vivo results demonstrate that Nab5822 CAR-T cells exhibit enhanced antitumor activity and no overt toxicity in the NSG xenograft model compared with monospecific CAR-T cells and Nab2258 CAR-T cells.

### Nab5822 CAR-T cells retain potent in vivo antitumor activity and safety in the presence of sBCMA

**Fig. 7:**
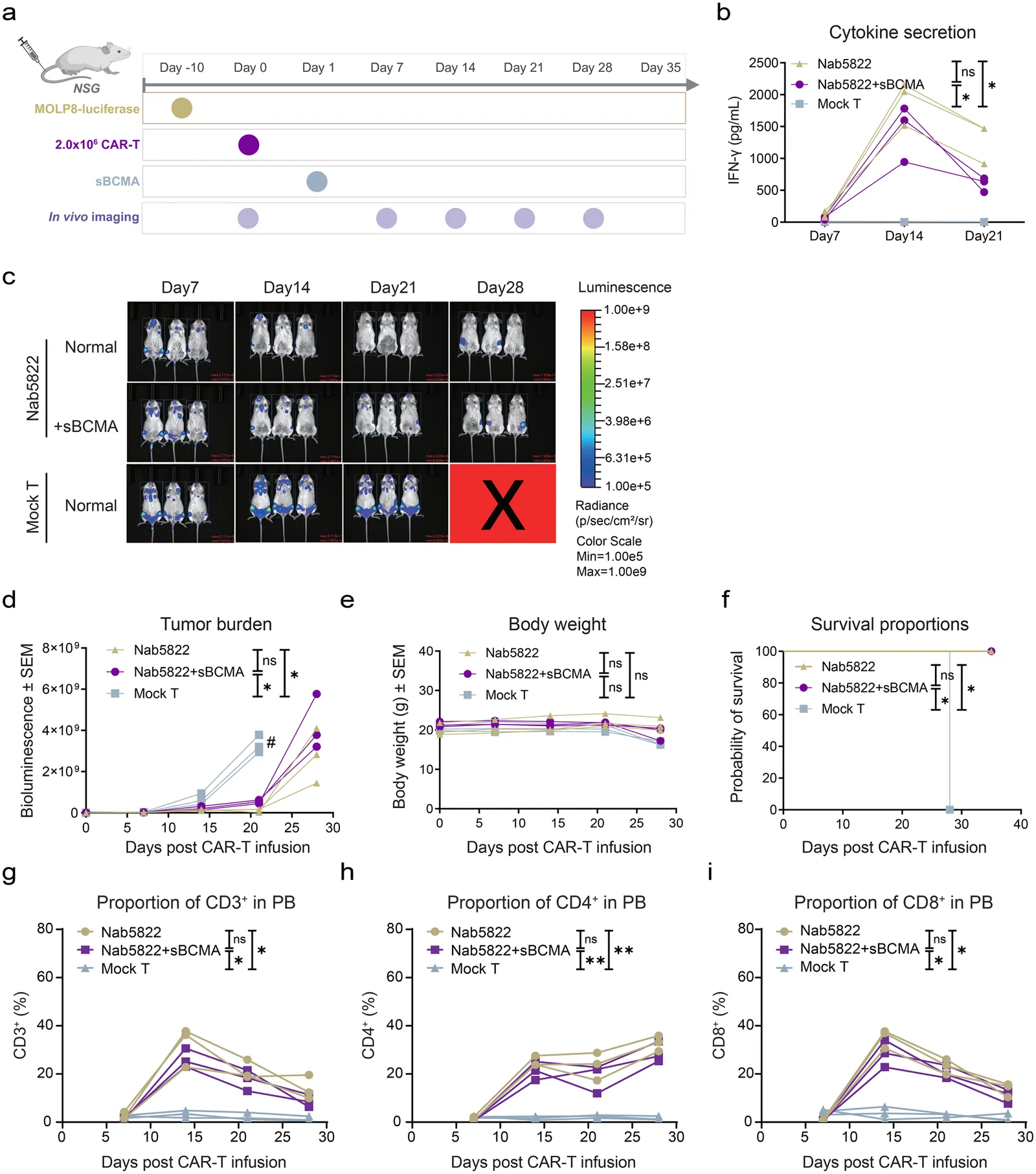
In vivo antitumor activity of Nab5822 CAR-T cells is preserved despite sBCMA. (**a**) Schematic of the evaluation of in vivo anti-BCMA^+^ tumor activity of Nab5822. NSG mice received 3.0 × 10^6 MOLP8-luc cells on day −10 and 2.0 × 10^6 cryopreserved Nab5822 CAR-T or mock T cells on day 0. Soluble BCMA (120 ng/mL) was administered on day 1, and tumor growth was monitored weekly. (**b**) IFN-γ concentrations were measured by ELISA on day 7 after infusion of Nab5822 CAR-T or mock T cells, in the absence or presence of sBCMA. (**c**) Total flux represents the tumor burden in MOLP8-luc bearing mice at the indicated time points (*n* = 3 biological replicates). (**d**) Bioluminescence was monitored during treatment. Mice marked with # were euthanized upon reaching the humane endpoint because tumor burden exceeded 1.0 × 10^10 photons/s. (**e**) in vivo toxicity was assessed by monitoring mouse body weight during treatment. Data are presented as mean ± SEM. (**f**) Kaplan–Meier survival curves of MOLP8-Luc bearing mice treated as described in (**c, d**). (**g-i**) Kinetics of CD3^+^ (**g**), CD4^+^ (**h**) and CD8^+^ (**i**) T cells in peripheral blood of mice after infusion of Nab5822 CAR-T or mock T cells. Each line represents an individual mouse. Statistical significance was determined by one-way ANOVA followed by Dunn’s multiple comparisons test, two-way ANOVA followed by Tukey’s multiple comparisons test, or log-rank test

To further evaluate the antitumor activity of Nab5822 CAR-T cells in the presence of sBCMA in vivo, we employed the same MM xenograft model in NSG mice as previously described. Mice were randomly assigned to control, normal, or sBCMA groups and received a single intravenous dose of Nab5822 CAR-T or mock T cells (2.0 × 10^6 cells). In the sBCMA group, recombinant sBCMA protein at 120 ng/mL was administered via tail vein injection on day 1 following CAR-T infusion as a supraphysiologic interference challenge, rather than as a direct surrogate for average patient serum levels. Tumor progression was monitored twice weekly by BLI, and mice were assessed daily for signs of toxicity, including changes in body weight, activity, or behavior (Fig. 7a). CAR-T cell expansion and persistence were assessed by quantifying the proportions of CD3^+^, CD4^+^, and CD8^+^ T cells in peripheral blood on days 7, 14, 21, and 28. Concurrently, mouse serum samples were analyzed for IFN-γ levels (Supplementary Fig. 9a).

Nab5822 CAR-T cells effectively secreted IFN-γ during treatment, regardless of sBCMA, with levels significantly higher than those in the mock T cells, suggesting that sBCMA does not impair the proinflammatory cytokine secretion capacity of Nab5822 CAR-T cells (Fig. 7b). Consistently, Nab5822 CAR-T cells exhibited superior antitumor efficacy compared with mock T cells in both the presence and absence of sBCMA (Fig. 7c–d and Supplementary Fig. 9b-e). Although sBCMA partially attenuated CAR-T function, no statistically significant differences were observed between the sBCMA and non-sBCMA groups (Supplementary Fig. 9b-e). Nab5822 CAR-T treated mice did not exhibit significant body weight loss or other adverse effects, indicating a favorable safety profile, consistent with previous observations (Fig. 7e). Kaplan-Meier survival analysis further demonstrated that Nab5822 CAR-T cells effectively prolonged survival compared with mock T cells, even in the presence of sBCMA (Fig. 7f).

Analysis of T cell expansion kinetics revealed that Nab5822 CAR-T cells proliferated effectively in the presence of sBCMA. Compared with day 7, the proportions of CD3^+^, CD4^+^, and CD8^+^ T cells increased significantly on days 14, 21, and 28 (Fig. 7g–i and Supplementary Fig. 9f-q). Notably, no statistically significant differences were observed between CAR-T cells administered with or without sBCMA at the corresponding time points, indicating that sBCMA did not measurably impair CAR-T-cell expansion or persistence in this NSG model (Supplementary Fig. 9f-q). Collectively, these findings provide a rationale for further clinical evaluation of Nab5822 CAR-T cells in relapsed or refractory multiple myeloma with elevated sBCMA.

### Non-overlapping epitope recognition underlies the biparatopic activity of Nab5822

**Fig. 8:**
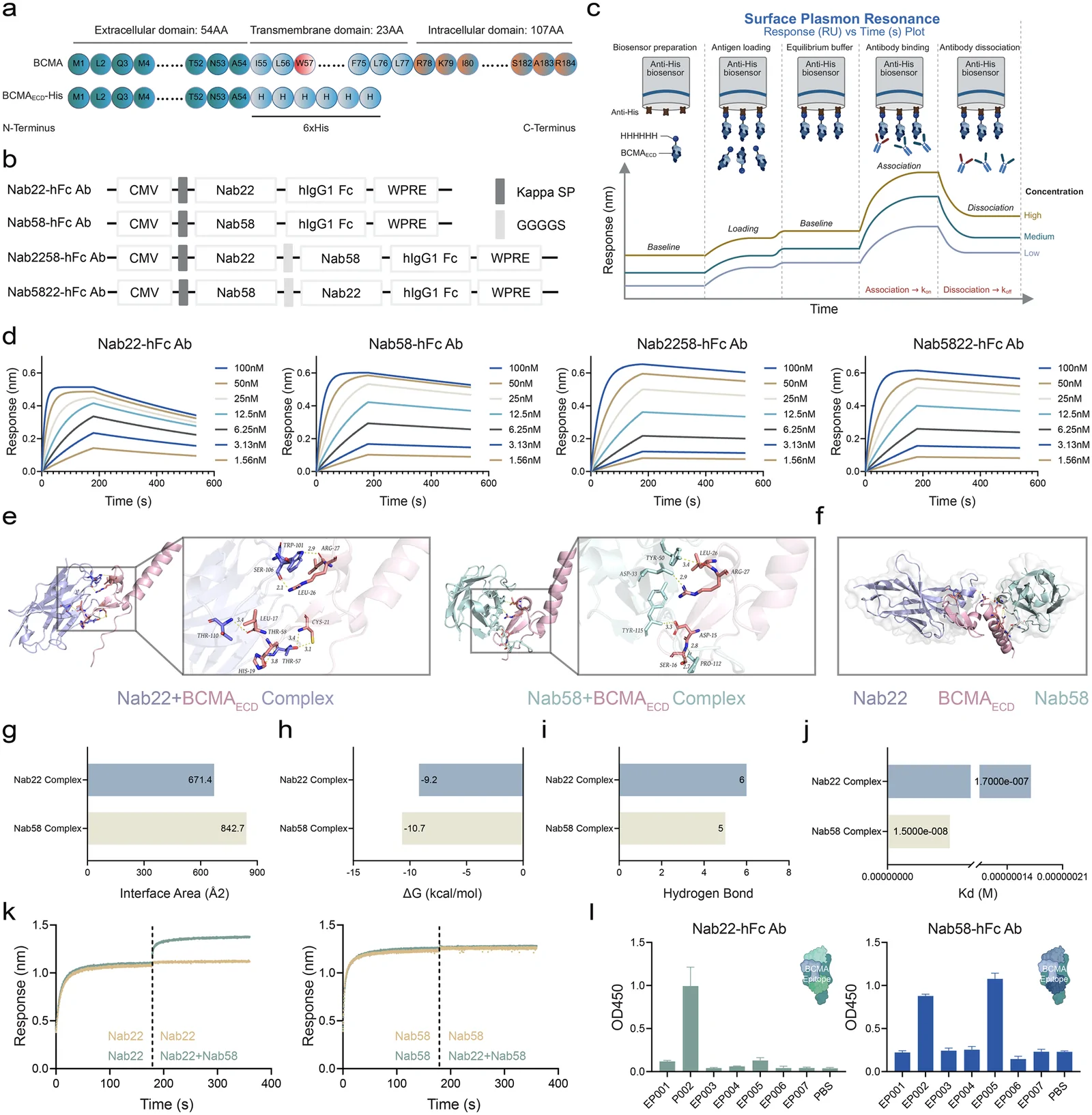
Distinct epitope recognition by Nab22 and Nab58 explains Nab5822 synergy. (**a-b**) Schematic diagrams of BCMA, BCMA_ECD_-His (**a**), and VHH-hFc (**b**) constructs used in this study. (**c**) Workflow of surface plasmon resonance (SPR) for real-time binding analysis. (**d**) Affinity of Nab22-hFc, Nab58-hFc, Nab2258-hFc, and Nab5822-hFc to BCMA as determined by SPR. (**e**) Model of the Nab22-BCMA complex (left) and Nab58-BCMA complex (right). (**f**) Superposition of the Nab22 and Nab58 complexes based on BCMA_ECD_. Residues contacting Nab22 are outlined in light blue, and those contacting Nab58 in light green. (**g-j**) Interface area (**g**), ΔG (**h**), hydrogen bond count (**i**), and Kd (**j**) were analyzed based on the antibody-antigen complexes. (**k**) Competition for BCMA binding between Nab22 and Nab58 was assessed by SPR. His-tagged human BCMA_ECD_ was immobilized on a anti-His sensor chip as the ligand. Nab22 (100 nM) or Nab58 (100 nM) was injected over BCMA_ECD_-His, followed by a mixture of Nab22 and Nab58. (**l**) Mapping of non-overlapping linear epitopes recognized by anti-BCMA antibodies Nab22 and Nab58

We hypothesized that the enhanced antitumor activity of Nab5822 arises from its bivalent design, which increases binding strength to target cells and may contribute to more efficient tumor elimination. To test this, we expressed and purified monovalent and bivalent tandem nanobodies and examined their interactions with recombinant BCMA_ECD_-his protein using SPR (Fig. 8a–c). With the exception of Nab22, Nab58, Nab2258, and Nab5822 exhibited comparable molar affinities (7.77 × 10^-10 M for Nab58, 7.47 × 10^-10 M for Nab2258, 6.22 × 10^-10 M for Nab2258) and similar association and dissociation kinetics (Fig. 8d and Supplementary Fig. 10a). Flow cytometry further confirmed broadly similar binding profiles across BCMA-heterogeneous cell lines. Although Nab5822 showed a trend toward lower EC_50_ values in some settings, the differences were modest (Supplementary Fig. 10b-c). These results suggest that the superior antitumor activity of Nab5822 is not fully explained by the measured equilibrium affinity alone, indicating that additional mechanisms likely contribute to CAR-T functionality. To explore potential structural differences, we generated three-dimensional homology models for all formats using SWISS-MODEL (Supplementary Fig. 10d). Computational analyses of melting temperature, isoelectric point (pI), and energy and charge under physiological conditions (0.15 M ionic strength, pH 2.0–8.0) revealed no significant differences among formats (Supplementary Fig. 10e-h).

Previous studies indicate that recognition of distinct epitopes can synergistically enhance CAR-T cell cytotoxicity, stabilize immune synapses, and improve persistence [39]. Based on this, we hypothesized that the biparatopic architecture of Nab5822 exploits non-overlapping epitope recognition to achieve superior antitumor activity. Molecular docking and dynamics simulations mapped the epitopes targeted by Nab22 and Nab58 on BCMA_ECD_. Docking analyses demonstrated that Nab22 and Nab58 recognize distinct epitopes with minimal overlap, with Nab22 binding the distal membrane region and Nab58 binding the proximal region (Fig. 8e–f). Quantitative comparison revealed that Nab58 forms a larger interface area (842.7 Å^2^) than Nab22 (671.4 Å^2^) and exhibits slightly weaker Gibbs free-energy change (ΔG = −10.7 kcal/mol vs −9.2 kcal/mol), indicating relative stability (Fig. 8g–h). The predicted number of hydrogen bonds was similar in both complexes, and equilibrium dissociation constants were consistent with previous experimental data (Fig. 8d and i-j).

Furthermore, SPR-based competitive binding assays showed that Nab22 and Nab58 interact with BCMA in a mutually exclusive manner (Fig. 8k), and linear peptide mapping confirmed non-overlapping epitope engagement (Fig. 8l and Supplementary Fig. 10i), further confirming their engagement of distinct epitopes. To further assess whether biparatopic engagement influences target-cell contact organization, we performed immunological synapse imaging and found that Nab5822 CAR-T cells formed more stable synapses with tumor cells than the monospecific CAR-T cells, supporting a role for improved synapse organization in the enhanced activity of Nab5822 (Supplementary Fig. 10j). Collectively, these data indicate that Nab22 and Nab58 recognize distinct BCMA epitopes and that biparatotic engagement promotes improved synapse organization, providing a mechanistic basis for the enhanced activity of Nab5822 CAR-T cells.

## Discussion

Adoptive T-cell therapy (ACT) has become a transformative treatment for haematological malignancies, as shown by the clinical success of CD19-directed CAR T-cell therapies since their first FDA approval in 2017 [40]. Additional targets for CAR T-cell therapy are rapidly emerging, notably BCMA. To date, more than 150 clinical trials have been registered worldwide, and two FDA-approved BCMA-directed CAR T-cell products, idecabtagene vicleucel and ciltacabtagene autoleucel, are available for patients with R/R MM [41, 42]. Nevertheless, despite encouraging antitumor activity in clinical trials, patients eventually relapse primarily due to the limited persistence of CAR-T cells, insufficient antigen density, or interference from sBCMA in the peripheral circulation [17, 43]. Addressing these barriers requires novel CAR designs that maintain durable activity despite antigen-heterogeneity and high sBCMA burden.

Previous strategies to overcome BCMA-related resistance include GSIs to limit sBCMA shedding, combinations with ICIs [24, 44], CARs incorporating chimeric co-stimulatory or switch receptors (CCRs/SCRs) [20, 45], and multispecific CAR designs to enhance functional avidity and limit antigen escape (Supplementary Fig. 11a) [46]. Building on this foundation, our study develops a nanobody-based, biparatopic CAR strategy (Supplementary Fig. 11b). Nanobodies provide distinct advantages for CAR engineering, including small size, high stability, manufacturing simplicity, and access to epitopes often inaccessible to scFv [47]. Unlike scFv-based tandem CARs, which can be prone to mispairing or suboptimal folding, VHH domains preserve structural integrity and maintain antigen-binding affinity while minimizing self-association [48]. They also share high sequence similarity to the human VH gene family III and exhibit low immunogenicity [48], thereby supporting reliable CAR T-cell performance.

In this study, we engineered a biparatopic BCMA-targeted CAR T-cell platform by integrating the VHHs Nab22 and Nab58, which recognize distinct, non-overlapping epitopes, into a single receptor. The resulting Nab5822 CAR-T cells demonstrated enhanced persistence and cytotoxicity, while maintaining functional activity despite sBCMA-mediated interference, and showed superior antitumor activity in vitro and in vivo compared with monospecific CARs. Nab5822 retained cytotoxicity even at supraphysiologic sBCMA concentrations of 120 ng/mL. These findings indicate relative tolerance to sBCMA-mediated functional inhibition in our experimental models, but do not demonstrate absence of binding to soluble BCMA. It also displayed enrichment of T_SCM_ subsets, reduced expression of exhaustion markers, and no evidence of overt antigen-independent hyperactivation. Using the same strategy, we previously developed a related biparatopic GPRC5D-directed CAR-T approach. In a phase 1 study, the GPRC5D-targeted product OriCAR-017 showed encouraging clinical activity in patients with MM (NCT05016778) [49]. In the present preclinical study, Nab5822 maintained a favorable safety profile, providing additional translational context for the broader biparatopic nanobody-based CAR platform.

A key mechanistic insight is that the superior activity of Nab5822 is not fully explained by binding affinity alone, as its equilibrium dissociation constants are comparable to those of the monospecific CARs. Instead, structural modeling, molecular docking, and epitope mapping indicate that Nab22 and Nab58 engage distinct BCMA regions, which may promote more stable immune synapse formation and more efficient signaling. Direct biophysical comparison of Nab5822 binding to soluble and membrane-associated BCMA was not performed in this study, and therefore the basis for its relative resistance to sBCMA-mediated functional inhibition remains to be defined. Moreover, BCMA alterations including R27P, P33S, S30del and P34del have been reported in patients with MM after BCMA-targeted T-cell engager (TCE) therapy, and molecular docking revealed that the key interacting residues of Nab22 and Nab58 do not involve amino acids 30, 33 or 34, suggesting potentially preserved activity against BCMA variants [50]. However, this inference is currently based on in molecular docking and lacks experimental validation, which is a limitation of the present study. Future work will validate the predicted interface through BCMA epitope mutagenesis and subsequent quantitative binding and functional assays. This biparatopic engagement drives coordinated phosphorylation of CD3ζ and STAT5 and sustains cytotoxicity, proliferation, and cytokine production under repeated antigen stimulation, without inducing antigen-independent activation. Together, these mechanisms provide a mechanistic basis for the robust in vivo activity of Nab5822.

A conceptual comparison with Nab2258 is instructive. The differential functional activity between Nab5822 and Nab2258 CAR-T cells may be explained by the spatial positioning of their targeted BCMA epitopes. Previous studies show that epitope-to-membrane distance critically affects immune synapse formation and the magnitude of CAR signaling [51, 52]. Docking analyses indicate that Nab22 binds a membrane-distal epitope, whereas Nab58 recognizes a membrane-proximal site. The biparatopic Nab5822 construct combines two non-overlapping domains and thereby exploits complementary epitope engagement across different membrane distances. This configuration likely stabilizes immune synapse formation and optimizes receptor orientation, thereby enhancing signalling efficiency [52]. In contrast, although Nab2258 incorporates the same nanobodies, the relative orientation and tandem arrangement of its binding domains may limit epitope accessibility and receptor clustering, leading to impaired synapse stability, attenuated signaling, and diminished functional activity [52, 53].

Our findings further support the clinical feasibility of this strategy. Nab5822 CAR-T cells generated from patient-derived PBMCs maintained robust antitumor activity despite impaired baseline fitness, thereby overcoming a common barrier to clinical translation. In contrast to existing BCMA-directed therapies, which often display early functional decline and limited persistence, Nab5822 preserved memory-like phenotypes and sustained function. These results suggest that biparatopic, nanobody-based CAR-T cells could prolong remission and reduce relapse, addressing a critical unmet need in MM.

Limitations should be acknowledged. First, our in vivo studies used immunodeficient NSG xenograft models, which cannot fully recapitulate the human bone marrow microenvironment or its associated immune interactions, and therefore may overestimate CAR-T-cell persistence while failing to capture clinically relevant toxicities. In addition, the current safety assessment was limited to body weight and observable signs of toxicity and did not include systemic cytokine profiling or histopathological evaluation. Second, although our tandem design performed well, evidence that tandem scaffolds accelerate receptor internalization suggests that linker architecture and spacing may need further optimization before clinical deployment. Third, we did not perform direct benchmarking against clinically validated BCMA CAR constructs, which precludes conclusions regarding relative performance versus established clinical products. Finally, although Nab5822 maintained activity under supraphysiologic sBCMA conditions in vitro and in vivo, the current models do not fully recapitulate the long-term effects of sustained sBCMA exposure in patients. Moreover, the in vitro assays were designed to model concurrent exposure of CAR-T cells to sBCMA and target cells, rather than a separately timed pre-incubation step, to better reflect the in vivo setting. Accordingly, the impact of different sBCMA pre-exposure durations was not formally evaluated, and because the functional assays relied on recombinant sBCMA rather than patient-derived serum or sBCMA, they do not fully capture the molecular complexity of patient samples or the potential influence of other soluble serum factors on CAR-T-cell activity.

Despite these limitations, the paradigm demonstrated here has important implications for ACT. Biparatopic nanobody-based CARs could be applied to other TAAs characterised by heterogeneous or low antigen density, conformational masking or susceptibility to soluble protein shedding, such as CD19, CD20 and CD22 in haematological malignancies, and MUC1, mesothelin and GPC3 in solid tumors [54, 55]. The favorable manufacturability and modularity of nanobody scaffolds also support their integration into advanced platforms, including allogeneic “off-the-shelf” CAR-T cells, in vivo delivery via lentiviral or LNP-mRNA vectors [56], and combinations with ICIs [24], γ-secretase inhibition [25], multispecific CAR [46], oncolytic virus (OVs) [57], and bispecific T-cell engagers (BiTEs) [58, 59]. Collectively, these applications underscore the versatility of nanobody-based designs and position Nab5822 as a prototype for resilient and scalable next-generation cellular therapies.

In conclusion, our study optimized BCMA CAR-T cells by incorporating two non-overlapping epitope-recognizing VHHs, validated through comprehensive in vitro and in vivo models. Biparatopic Nab5822 CAR-T cells showed superior antitumor efficacy in vitro and in murine tumor models with low antigen density, while maintaining functional activity despite sBCMA-mediated interference. These findings provide a blueprint for next-generation, nanobody-based biparatopic CAR-T therapies and support further preclinical and translational evaluation in MM, while direct benchmarking against clinically validated BCMA CAR constructs remains an important next step. Larger trials are required to define its efficacy, safety and potential integration into combination strategies, particularly with GPRC5D-targeted or other immune- and molecularly directed therapies.

## Methods

### Design of the study

This study aimed to (i) identify the optimal candidate for BCMA-targeted immunotherapy and (ii) develop an optimized bispecific CAR for MM treatment, with the goals of enhancing in vivo persistence and antitumor activity, effectively targeting BCMA-low tumor cells, and mitigating the impact of sBCMA. BCMA-targeted CARs were generated through screening of a humanized VHH phage display library. Their efficacy and safety were rigorously evaluated using in vitro functional assays and murine xenograft models. By integrating two VHHs that recognize non-overlapping extracellular BCMA epitopes, we hypothesized that the bispecific CAR-T design would improve functional activity, T-cell activation, and persistence compared with conventional monospecific CAR-T cells. We anticipated that this design would allow effective targeting of BCMA-low tumor cells, reduce sBCMA interference, and enhance both in vivo persistence and antitumor efficacy.

### Human BCMA expressing cell line generation

The full-length sequence of human BCMA (NBCI Accession: NM_001192.2) was purchased from Sino Biological (China, HG10620-M). The sequences were cloned into the lentiviral vector pCore-fEF1a-MCS-IRES-GFP and co-transfected into HEK293 cells (CRL-3216, ATCC) along with the helper plasmids pMDLg/pRRE, pRSV-REV, and pVSVG. The lentivirus-containing supernatant was harvested after determining the viral titer. Chinese hamster ovary (CHO) and Human embryonic kidney (HEK) 293T cells were cultured in Dulbecco’s Modified Eagle Medium (DMEM, Gibco) supplemented with 10% Fetal Bovine Serum (FBS, Gibco) at 37 °C, 5% CO_2_. When the cells reached 80% confluence, CHO and HEK293 cells were incubated with the lentivirus for 24 hours. Transfection efficiency was verified by flow cytometry analysis of GFP expression. Subsequently, CHO and HEK293T cells were transduced with the lentivirus to stably express human BCMA, and single-cell cloning was performed to establish the CHO-hBCMA and HEK293T-hBCMA cell lines.

### Quantification of BCMA density

BCMA molecules on the surface of MM cell lines were quantified using the Quantibrite^TM^ PE Fluorescence Quantitation Kit (BD Biosciences, #340495), following the manufacturer’s protocol. This method converts PE geometric mean fluorescence intensity (gMFI) values per cell into absolute numbers of binding sites by applying established PE-to-antibody ratios. Data acquisition was performed on a BD FACSCelesta^TM^ flow cytometer, and results were analyzed with FlowJo software.

### Cell lines and culture condition

Cell lines NCIH929, MM.1S, MOLP8, and K562 were procured from either the American Type Culture Collection (ATCC) or the Cell Bank of the Chinese Academy of Sciences (CAS). NCIH929, NCIH929-BCMA^KO^, MM.1S, MOLP8, and K562 cells were genetically modified to express the fusion protein firefly luciferase. The cells were cultured using RPMI1640 medium (Gibco) supplemented with 10% FBS. The presence of viruses and mycoplasma in all cell lines is routinely assessed monthly using quantitative polymerase chain reaction (qPCR). Additionally, all cell lines were maintained at 37 °C and 5% CO_2_.

### Humanized phage display synthetic nanobody library generation

The full-length sequence of Caplacizumab were synthesized by GENEWIZ (China), and cloned into the HP153 phagemid vector. A humanized Caplacizumab-derived VHH scaffold was selected as the library framework because of its favorable developability properties, including stable folding, robust expression, and suitability as a clinically relevant backbone. Sequence diversity was introduced specifically into the complementarity determining regions (CDRs) by synthetic oligonucleotide design and Kunkel mutagenesis [60]. Whereas framework residues were preserved to maintain proper VHH folding and structural integrity. In designing the synthetic oligonucleotides, the main considerations were to maximize paratope diversity for broad nanobody discovery while minimizing sequence liabilities that could compromise library quality and downstream developability. These liabilities included premature stop codons, excessive hydrophobicity, framework destabilization, and residues likely to increase aggregation risk. Double strand DNA was transformed into M13KO7 helper phage pre-infected competent *E.coli SS320 c*ells by electroporation. The phage-containing supernatant was collected after over-night culture and stored in aliquots of glycerol stocks at −80 °C. The diversity of this library is 1.0 × 10^10.

### Whole-cell panning

For BCMA biopanning, the humanized nanobody library was subjected to two rounds of selection on CHO-hBCMA cells, followed by two additional rounds on HEK293T-hBCMA cells. ~1.0 × 10^13 phage particles were blocked in 5% FBS diluted by PBS, rotating for 1 h at 4 °C. 1.0 × 10^7 cells were collected, washed by PBS, and blocked in 5% FBS diluted by PBS incubating at 4 °C for 1 h. The blocked phage and cells were gently mixed and incubated rotating for 2 h on ice. The cells were then washed three times with PBS, and the phage was eluted by 100 nM Gly-HCl (pH2.2) for 10 min at room temperature. After centrifugation the supernatant was neutralized with 2 M Tris (pH8.0), and the phage eluate was used to infect TG1 cells in the logarithmic growth phase. Phage particles were prepared for the next round of biopanning by amplification and rescue with M13K07 helper phage [61]. Each round of panning was pressurized by increasing washing times and reducing cells number and binding times until phage was specifically enriched to BCMA.

### Monoclonal phage ELISA

Individual phage clones from the third or fourth biopanning round were evaluated for binding activity to target cells by protein- or cell-based Enzyme Linked Immunosorbent Assay (ELISA). Ultimately, BCMA-specific nanobodies were finally obtained by sequencing the positive clones.

### Expression and purification

All nanobodies were cloned into the pcDNA3.4-human IgG1 Fc plasmid and transfected into EXPI293F cells using polyethyleneimine (PEI), then expressed as VHH-hFc fusions and purified via Protein A affinity chromatography. Specifically, they were bound to a protein A column in 1×PBS (Hyclone), eluted with 100 mM Gly-HCl (pH 3.0), and neutralized with 1 M Tris (pH 8.0). Finally, the purified nanobodies were quantified via a BCA Protein Assay Kit (Thermo Scientific). BCMA_ECD-_His (residues 1–54 of human BCMA, UniProt: Q02223) was synthesized, constructed, and expressed by Oricell Therapeutics.

### SEC-HPLC

Analysis of Size-exclusion chromatography (SEC) was performed using an Agilent-1200 high-performance liquid chromatography system (HPLC) recommended by the manufacturers. Briefly, Nanobodies were diluted and injected into SEC-300 columns. The elution of each dAb was monitored at 280 nm using an Agilent-1200 series UV Absorbance detector.

### Surface plasmon resonance, SPR

To assess the binding affinity of the VHHs obtained in this study, we performed surface plasmon resonance (SPR) analysis using the Octet RED384 System (Sartorius). The BCMA extracellular domain with a His tag (BCMA_ECD_-His) was diluted to 100 nM and immobilized on Anti-His biosensors (Sartorius) for 120 s. Serially diluted VHH-hFc nanobodies were then applied, with an association time of 180 s and a dissociation time of 360 s. Between cycles, the sensor surfaces were regenerated by injecting 10 mM glycine (pH 2.0) for 120 s. The dissociation constant (*KD*) values were determined using Octet Data Analysis 10.0 software.

### Construction of lentiviral vectors

The nanobody sequences were subcloned into the lentiviral plasmid pCore-BBζ (Oricell Therapeutics), which comprises the human EF-1α promoter, signal peptide, CD8α hinge, CD8α transmembrane domain, 4-1BB co-stimulatory domain, and CD3ζ signaling domain. For the bispecific CAR, the two nanobodies were expressed with each nanobody connected via a flexible linker. The plasmid construct was transformed into DH5α *E. coli* and propagated for plasmid extraction using a Midiprep Kit. The fidelity of the inserted sequences was confirmed by Sanger sequencing.

### Production of lentivirus

Lentivirus was produced by co-transfecting HEK293T cells with a four-plasmid system that included the helper plasmids pMDLg/pRRE, pRSV-REV, and pVSVG. After 48 hours, the supernatants containing lentivirus particles were harvested, filtered through a 0.45 µm filter, and stored at −80 °C. The viral titer in transduction units per milliliter (TU/mL) was determined by analyzing the transduced HEK293T cells using flow cytometry.

### Generation of CAR-T cells

Human primary CD3^+^ T cells were isolated from cryopreserved peripheral blood mononuclear cells (PBMCs) using the Miltenyi CD3^+^ T-cell isolation kit (Miltenyi Biotec, 130–050-101). PBMCs used for CAR-T-cell preparation were obtained either from healthy donors or from patients with relapsed or refractory multiple myeloma. For patient-derived samples, PBMCs were collected by leukapheresis at the hospital and transported to the manufacturing center for CAR-T-cell production before cryopreservation. CD3^+^ T cells were then activated with anti-CD3/CD28 Dynabeads (Gibco, 40203D) and incubated in X-VIVO [15] medium (Lonza) containing 5% FBS and 200 U/mL recombinant human interleukin-2 (IL-2) for 24 hours. The activated T cells were transduced with lentivirus at a multiplicity of infection (MOI) of 3, followed by centrifugation at 1200 rpm for 60 min. After transduction, CAR-T cells were counted and subcultured every 2 days at a density of 0.5–1.0 × 10^6 cells/mL. Dynabeads were removed from the culture system after 5 days of co-culture.

### Flow cytometry analysis

For flow cytometry assays, cells were washed and resuspended in FACS buffer (PBS with 2% FBS). CAR-T cell surface staining for CAR molecules was performed with PE-conjugated CD3 and APC-labeled BCMA_ECD_-His protein. For CD4/CD8 T-cell subset analysis, cells were stained with BV421-conjugated CD3, BV510-conjugated CD4, and APC-Cy7-conjugated CD8. Detection of activation markers was carried out using BV510-conjugated CD3, PE-conjugated CD137 (4-1BB), PE-Cy7-conjugated CD25, and APC-conjugated CD69. Exhaustion markers were evaluated with APC-conjugated CD3, BV421-conjugated PD1, BV510-conjugated TIM3, and PE-conjugated LAG3. Stem cell memory T cells were identified with PE-conjugated CCR7, BV510-conjugated CD45RA, and PE-Cy7-conjugated CD95. Perforin and granzyme analysis was performed with BV421-conjugated CD3, PerCP-Cy5.5-conjugated CD8, Alexa Fluor488-perforin, and DyLight650-Granzyme B. For intracellular phospho-signaling assessment, CAR-T cells were co-cultured with MM cells, harvested at 15 and 30 minutes after antigen exposure, fixed with pre-warmed formaldehyde, permeabilized with methanol, and stained with antibodies against phosphorylated CD3ζ and STAT5. All data were acquired with a FACSCelesta™ flow cytometer and analyzed with FlowJo software.

### NFAT reporter assay

Jurkat-NFAT-GFP reporter cells were generated in-house and harbour a GFP reporter driven by a nuclear factor of activated T cells (NFAT)-responsive promoter. Reporter cells expressing the indicated CAR constructs were co-cultured with or without BCMA-positive MM.1S cells. GFP fluorescence was quantified by flow cytometry as a measure of basal and target-induced NFAT activation. Mock Jurkat-NFAT-GFP cells were used as the background control.

### Cell apoptosis assay

Apoptotic cell death was evaluated using the Pacific Blue^TM^ Annexin V/PI Apoptosis Detection Kit (BioLegend, 640928) following the manufacturer’s protocol, and analyzed by flow cytometry.

### Cytotoxicity assay

Cytotoxicity assays were performed based on bioluminescence measurements. Briefly, CAR-T cells were incubated with luciferase-expressing target cells at varying effector-to-target (E:T) ratios in the absence or presence of recombinant BCMA_ECD_-His protein for 24 hours (short-term cytotoxicity, and sBCMA experiment) at 37 °C. K562-luciferase cells were used as the negative control cells. After incubation, the culture medium was gently mixed and approximately 50 μL per well was added to each well. The luciferase substrate (Promega, #E2610) was subsequently added under dark conditions. The relative luminescence units were measured using the Microplate Reader according to the provided instructions. Cytotoxicity was calculated using the following formula:

$${{{\rm{Total\;target\;cell\;luminescence}} - {\rm{luminescence\;of\;remaining\;cells\;after\;lysis}}} \over {{\rm{Total\;target\;cell\;luminescence}}}}{\rm{}} \times {\rm{}}100{\rm{\% }}$$

### Degranulation assay

To evaluate CD107a expression on the cell surface, CAR-T cells were co-cultured with MM.1S or NCIH929 cells at a 1:1 ratio in 12-well plates containing 1.2 mL X-VIVO [15] medium. Anti-CD107a-BV510 antibody (BD Biosciences, #563078) and GolgiStop^TM^ protein transport inhibitor (BD Biosciences, #554724) were added at the start of the 4 hour incubation at 37 °C. Cells were then harvested and stained with anti-CD3-APC and anti-CD8-PerCP-Cy5.5. The percentage of CD107a expression in CD3^+^ CD8^+^ T cells was detected using flow cytometry, and the results were analyzed with FlowJo software.

### Immunological synapse formation assay

CAR-T cells were co-cultured with MM.1S target cells at a 1:1 ratio for 10 min and then transferred to Nunc chamber slides (Thermo Scientific) precoated with ICAM-Fc (1 μg/mL; R&D). Cells were fixed with 4% paraformaldehyde, blocked with 5% horse serum, and stained with anti-γ-tubulin antibody (clone GTU-88; Sigma) to assess centrosome polarization. After washing, cells were incubated with Alexa Fluor 488-conjugated secondary antibodies together with anti-CD8-PE (SouthernBiotech, 1550-09 L) and NucRed 647 nuclear stain (Invitrogen, R37113). Cells were then washed, mounted and analysed by confocal microscopy. We quantified the polarization level of the microtubule organizing center (MTOC), a crucial component in maintaining the stability of the immune synapse, a critical step in CAR-T therapy.

### Cytokine release assay

4.0 × 10^4 CAR-T cells were cultured alone or co-cultured with 4.0 × 10^4 target cells (at a 1:1 ratio) in 0.2 mL of X-VIVO [15] medium without added cytokines in a 96-well plate for 24 hours. In sBCMA experiments, CAR-T cells were co-cultured with 4.0 × 10^4 target cells at a 1:1 ratio in the presence of recombinant BCMA_ECD_-His protein for 24 hours. Recombinant sBCMA was present throughout the co-culture period rather than applied as a separately timed pre-incubation step. After culture completion, the plates were centrifuged at 250×g for 5 minutes. Supernatants were collected and analyzed for interleukin-2 (IL-2), tumor necrosis factor (TNF-α), and interferon-γ (IFN-γ) using a Cytometric Bead Array (CBA) Kit (BD Biosciences, #550749), following the manufacturer’s instructions. Supernatants were diluted 2 to 5 times in the recommended buffer, and cytokine concentrations were determined based on the standard curve and adjusted for the dilution factor.

### Repeated antigen stimulation assay

Ex vivo expansion and persistence of CAR-T cells were assessed by co-culture with MM cells, with the cells harvested and re-cultured every 24 hours. Briefly, CAR-T cells and MM target cells were co-cultured at a 1:1 ratio in X-VIVO [15] medium, either in the absence or presence of recombinant sBCMA at 12 ng/mL, corresponding to the mean serum concentration measured in our patient cohort. After 24 hours, co-cultured cells were harvested, washed with 1×PBS, resuspended in fresh X-VIVO [15] medium, and added to fresh MM cells for re-culture under the same conditions. The numbers and proportions of CAR-T and tumor cells were determined using AO/PI reagent to distinguish live and dead cells, and by flow cytometry to quantify cell populations. This procedure was repeated for each subsequent co-culture cycle.

### Animal studies

All animal procedures were conducted by certified personnel following protocols approved by the Animal Research Committee of Shanghai YouShuLife Technology Co., Ltd. Mice were maintained under specific pathogen-free (SPF) conditions with controlled temperature and humidity. Five- to eight-week-old female NSG mice (NOD.Cg-Prkdc^scid Il2rg^tm1Wjl) were intravenously injected with 2.0 × 10^6 MOLP8-luciferase cells via the tail vein. Cryopreserved CAR-T cells and mock T cells were administered via tail vein injection once the mean tumor bioluminescence reached 2.0 × 10^6 photons/sec. CAR expression levels were normalized across all CAR-T cell groups by adjusting the proportion of CAR-positive cells through the addition of mock T cells prior to infusion. For the sBCMA interference study, recombinant sBCMA protein (120 ng/mL) was administered via tail vein injection on day 1 after CAR-T infusion. Bioluminescence imaging (BLI) was performed twice weekly, and mice were monitored daily for signs of toxicity, including weight loss, lethargy, or abnormal behavior. Blood samples were collected weekly from the tail vein to assess CAR-T cell levels in NSG mice. Cytokine levels were measured using CBA kits at 7-day intervals after CAR-T infusion. Mice were euthanized according to protocol guidelines if they exhibited more than 20% body weight loss or a mean tumor bioluminescence of 1.0 × 10^10 photons/sec.

### Statistical analysis

All statistical analyses and data displays were performed using GraphPad Prism software, version 10.0. The statistical significance of in vitro and in vivo assays was determined using one-way ANOVA with Dunn’s multiple comparisons, two-way ANOVA with Tukey’s multiple comparisons, or an unpaired t-test. Mouse survival was analyzed using the log-rank test. Statistical analysis was performed after assessing data normality (Shapiro-Wilk test) and homogeneity of variance (Brown-Forsythe test). The significance of the findings is defined as follows: NS, not significant; *p < 0.05, **p < 0.01, ***p < 0.001, ****p < 0.0001.

## Electronic supplementary material

Below is the link to the electronic supplementary material.

Supplementary material 1

## Data Availability

The authors confirm that the data supporting the findings of this study are available within the article and its supplementary materials. The datasets generated or analyzed during the current study are available from the corresponding author upon reasonable request.
