# Supplementary material for: Humanized biparatopic nanobody-based CAR-T cells overcome antigen-heterogeneity in multiple myeloma
Source: J Transl Med. 2026 Jun 29;24:1191. doi: 10.1186/s12967-026-08533-z (PMC13584338; doi:10.1186/s12967-026-08533-z)
Supplement: Supplementary file 1 [file 12967_2026_8533_MOESM1_ESM.docx]

**Supplementary information**

Humanized biparatopic nanobody-based CAR-T cells overcome antigen-heterogeneity in multiple myeloma

Jincai Zhou^1†✉^, Kai Wu^1†^, Bixia Lei^1†^, Xinran Luo^1^, Feifei Shi^1^, Yuting Zhang^1^, Xuefeng Kong^1^, Huajing Wang^1^, Joy Zhou^1^, Hao Guo^1^, Xiaowen He^1✉^

**Supplementary Figure 1**

**Supplementary Figure 1. Purity and affinity characterization of anti-BCMA VHHs.** (a) Domain architecture and γ-secretase cleavage site of human BCMA. Schematic of the 184-aa BCMA protein showing the 54-aa extracellular domain, 23-aa transmembrane domain, and 107-aa intracellular domain. γ-Secretase cleaves at Trp57, releasing the soluble BCMA_ECD_. (b) Structural model of the human BCMA extracellular domain. (c-d) Polyclonal analysis of dilutions of the phage pool from each round of biopanning by cell-based ELISA using CHO-hBCMA and HEK293T-hBCMA cells. (e) Purity of candidate VHH antibodies was assessed by SDS-PAGE under reducing and non-reducing conditions. (f) Summary of HPLC-SEC and SDS-PAGE analyses of candidate VHH antibodies. Monomer purity is presented as a percentage. (g) Summary of binding kinetics of candidate VHH antibodies to BCMA_ECD_ determined by SPR. K_D_: equilibrium dissociation constant. K_D_ = K_OFF_/K_ON_. K_ON_: association rate constant. K_OFF_: dissociation rate constant.

**Supplementary Figure 2**

**Supplementary Figure 2. Specificity characterization of anti-BCMA VHHs.** (a-l) Flow cytometry analysis confirmed the specificity of candidate VHH antibodies, with no binding detected to BCMA-negative cell lines. Data were acquired on a BD FACSCanto II and analyzed using FlowJo software.

**Supplementary Figure 3**

**Supplementary Figure 3. *In vitro* expansion, viability, and CD4/CD8 composition of anti-BCMA VHH CAR-T cells.** (a) Fold expansion of VHH CAR-T cells cultured in vitro following anti-CD3/CD28 stimulation at the indicated time points. (b) Viability of VHH CAR-T cells over time following anti-CD3/CD28 stimulation. (c) The volume of CAR-T cells at different time points during culture, calculated as V=4πr^3^/3. (d) Representative flow cytometry analysis of CD4^+^ and CD8^+^ T cell populations. (e) Percentages of CD4^+^ and CD8^+^ T cell subsets in CAR-T and mock T cells.

**Supplementary Figure 4**

**Supplementary Figure 4. Antitumor activity of anti-BCMA VHH CAR-T cells.** (a-c) Cytotoxicity of CAR-T and mock T cells against MOLP8 (BCMA^Low^) (a), MM.1S (BCMA^Mid^) (b), and NCIH929 (BCMA^High^) (c) target cells at E:T ratios of 3:1, 1:1, and 1:3 in the presence of sBCMA. Data are presented as mean ± SD. (d-f) Cytotoxicity of six VHH CAR-T cells and mock T cells against MOLP8, MM.1S, and NCIH929 target cells at E:T ratios of 3:1, 1:1, and 1:3. (g-l) Cytokine production was measured by ELISA for IFN-γ and IL-2 in supernatants collected after co-culture at indicated E:T ratios. Data are presented as mean ± SD. Mock T cells served as negative controls. Statistical significance was determined by one-way ANOVA, followed by Dunn's multiple comparisons test; ns, not significant; *P < 0.05, **P < 0.01, ***P < 0.001, ****P < 0.0001.

**Supplementary Figure 5**

**Supplementary Figure 5. Antitumor activity of biparatopic CAR-T cells.** (a-b) Cytotoxicity of CAR-T and mock T cells against MOLP8 (BCMA^Low^), MM.1S (BCMA^Mid^), NCIH929 (BCMA^High^), and NCIH929-BCMA^KO^ (BCMA^Neg^) target cells at E:T ratios of 3:1, 1:1, and 1:3, in the absence or presence of sBCMA. Data are presented as mean ± SD. (c-d) Representative heat maps illustrating cytokine production of CAR-T and mock T cells against MOLP8, MM.1S, NCIH929, and K562 target cells at E:T ratios of 1:1, measured by CBA, are shown in the top panels without sBCMA and in the bottom panels with sBCMA.

**Supplementary Figure 6**

**Supplementary Figure 6. BCMA CAR constructs induce basal and target-dependent NFAT activation.** (a) Schematic overview of the NFAT-GFP reporter assay. In-house generated Jurkat-NFAT-GFP reporter cells expressing BCMA CAR constructs were incubated with BCMA-positive MM cells. Upon CAR recognition of BCMA antigen, downstream signaling activates NFAT and induces GFP expression as a measure of reporter activation. (b) Jurkat-NFAT-GFP reporter cells expressing the indicated BCMA CAR constructs were cultured alone or co-cultured with BCMA-positive MM.1S cells. Mock Jurkat-NFAT-GFP cells were included as a background control. CAR reactivity was assessed by flow cytometric measurement of GFP mean fluorescence intensity (MFI). (c-d) Representative flow cytometry analysis of CAR-T cell degranulation capacity, showing CAR-T cells co-cultured with MM.1S (c) or NCIH929 (d).

**Supplementary Figure 7**

**Supplementary Figure 7. Antitumor activity of Nab5822 CAR-T cells generated from PBMCs of three patient samples.** (a-b) Cytotoxicity of CAR-T and mock T cells against NCIH929 (BCMA^High^) (a) and MM.1S (BCMA^Mid^) (b) target cells at E:T ratios of 3:1, 1:1, and 1:3. Data are presented as mean±SD of triplicate wells. (c-e) IL-2 concentrations in supernatants collected after co-culture with NCIH929 (c) and MM.1S (d) at an E:T ratio of 1:1, including autocrine production (e). (f-h) IFN-γ concentrations in supernatants collected after co-culture with NCIH929 (f) and MM.1S (g) at an E:T ratio of 1:1, including autocrine production (h). CAR-T cells were generated from patient-derived PBMCs obtained from patients with relapsed or refractory multiple myeloma. Data are presented as mean ± SD.

**Supplementary Figure 8**

**Supplementary Figure 8. Memory differentiation, apoptosis, and signaling of biparatopic CAR-T cells.** (a-c) CAR-T and mock T cells were separately co-cultured with tumor cells over successive rounds of repeated tumor challenge in the presence of 12 ng/mL sBCMA, with cytotoxicity (a), CD3^+^ T cell numbers (b), and CAR-T cell numbers (c) quantified after each round. (d) Representative dot plots showing CCR7 and CD45RA expression in CD4^+^, CD8^+^, and CAR^+^ T cells. T cells were classified into stem cell memory (T_SCM_, CCR7^+^/CD45RA^+^), central memory (T_CM_, CCR7^+^/CD45RA^-^), effector memory (T_EM_, CCR7^-^/CD45RA^-^), and effector (T_EFF_, CCR7^-^/CD45RA^+^) subsets. (e) Quantification of T cell subsets shown in (d). Frequencies of T_SCM_, T_CM_, T_EM_, and T_EFF_ cells are plotted for CD4^+^, CD8^+^, and CAR^+^ T cells. (f-g) Frequencies of apoptotic CAR-T cells, measured by Annexin V (f) and PI (g), on day 12 after T-cell activation (n=3). (h-i) Phosphorylation of CD3ζ (h) and STAT5 (i) in CAR-T cells after co-culture with tumor cells. CAR T cells were serum-starved for 24 h and co-cultured with MM.1S cells at a 1:1 E:T ratio for 15 or 30 min. (j) Representative histograms showing the kinetics of CD3ζ phosphorylation in CAR-T cells after 15 or 30 min of co-culture with MM.1S cells, under the conditions shown in (h). (k) Representative histograms showing the kinetics of STAT5 phosphorylation in CAR-T cells after 15 or 30 min of co-culture with MM.1S cells, under the conditions shown in (i). Statistical significance was determined by one-way ANOVA, followed by Dunn's multiple comparisons test; ns, not significant; *P < 0.05, **P < 0.01, ***P < 0.001, ****P < 0.0001.

**Supplementary Figure 9**

**Supplementary Figure 9. Evaluation of the antitumor activity of biparatopic CAR-T cells *in vivo*.** (a) Schematic diagram illustrating the *in vivo* pharmacodynamics of CAR-T cells. Blood was collected from NSG mice via the tail vein on days 7, 14, 21, and 28. Following centrifugation, plasma was analyzed for cytokines, and T-cell expression levels were assessed. (b-e) Tumor burden was monitored by bioluminescence on days 7 (b) , 14 (c), 21 (d), and 28 (e), with data presented as mean ± SD. (f-i) Percentages of CD3^+^ T cells in peripheral blood were measured on days 7 (f) , 14 (g), 21 (h), and 28 (i), with data presented as mean ± SD. (j-m) Percentages of CD4^+^ T cells in peripheral blood were measured on days 7 (j) , 14 (k), 21 (l), and 28 (m), with data presented as mean ± SD. (n-q) Percentages of CD8^+^ T cells in peripheral blood were measured on days 7 (n) , 14 (o), 21 (p), and 28 (q), with data presented as mean ± SD. Statistical significance was determined by one-way ANOVA, followed by Dunn's multiple comparisons test; ns, not significant; *P < 0.05, **P < 0.01, ***P < 0.001, ****P < 0.0001.

**Supplementary Figure 10**

**Supplementary Figure 10. Evaluation of the properties of Nab22, Nab58, and Nab5822.** (a) Summary of the binding kinetics of monospecific and bispecific nanobodies to BCMA_ECD_ , determined by SPR. K_D_: equilibrium dissociation constant. K_D_ = K_OFF_/K_ON_. K_ON_: association rate constant. K_OFF_: dissociation rate constant. (b) Summary of EC_50_ values of monospecific and bispecific nanobodies against target cells with heterogeneous BCMA expression, determined by flow cytometry. (c) Flow cytometry analysis of the binding of Nab22-hFc, Nab58-hFc, Nab2258-hFc, and Nab5822-hFc to target cells with heterogeneous BCMA expression. (d) Modeling of Nab22, Nab58, and Nab5822 using SWISS-MODEL. Surface electrostatics are shown with blue indicating positive charge and red indicating negative charge. (e) Energies of Nab22, Nab58, and Nab5822 at 0.15M ionic strength across pH 2.0-8.0, calculated using the Protein-Sol webserver. (f) Charges of Nab22, Nab58, and Nab5822 at 0.15M ionic strength across pH 2.0-8.0, calculated using the Protein-Sol webserver. (g) Melting temperatures of Nab22, Nab58, and Nab5822. (h) Isoelectric points (pI) of Nab22, Nab58, and Nab5822. (i) Sequences and positions of linear BCMA_ECD_ epitope peptides. The structural delineation of BCMA_ECD_ is based on its disulfide bonds and secondary structure: three disulfide bonds (Cys-Cys) are formed between residues 8-21, 24-37, and 28-41, and the secondary structure from the N-terminus to the C-terminus comprises β-strands (residues 12-15, 20-23, 30-32), helices (residues 24-27, 35-37), and turns (residues 16-19, 38-40, 42-44). (j) Quantification of microtubule organizing center (MTOC) to synapse distance.

**Supplementary Figure 11**

**Supplementary Figure 11. Design of BCMA-specific CARs for multiple myeloma.** (a) To minimize BCMA low-to-negative escape, several new CAR designs have been proposed, including a BCMA CAR co-expressed with a CD38 chimeric co-stimulatory receptor (CCR), a tandem CAR engaging both CD38 and BCMA, the use of γ-secretase inhibitors (GSIs) to block shedding of sBCMA, and a BCMA HIT receptor (HLA-independent T cell receptor). (b) Nab5822 is a novel humanized biparatopic CAR incorporating 4-1BB and CD3ζ signaling domains and comprising two VHH-based domains that bind non-overlapping BCMA epitopes, thereby enhancing overall binding affinity and T cell avidity.
